# RFfiller: a robust and fast statistical algorithm for gap filling in draft genomes

**DOI:** 10.7717/peerj.14186

**Published:** 2022-10-14

**Authors:** Firaol Dida Midekso, Gangman Yi

**Affiliations:** Department of Multimedia Engineering, Dongguk University, Seoul, South Korea

**Keywords:** Gap filling, DNA sequencing, Read extension, De novo assembly

## Abstract

Numerous published genomes contain gaps or unknown sequences. Gap filling is a critical final step in de novo genome assembly, particularly for large genomes. While certain computational approaches partially address the problem, others have shortcomings regarding the draft genome’s dependability and correctness (high rates of mis-assembly at gap-closing sites and high error rates). While it is well established that genomic repeats result in gaps, many sequence reads originating from repeat-related gaps are typically missed by existing approaches. A fast and reliable statistical algorithm for closing gaps in a draft genome is presented in this paper. It utilizes the alignment statistics between scaffolds, contigs, and paired-end reads to generate a Markov chain that appropriately assigns contigs or long reads to scaffold gap regions (only corrects candidate regions), resulting in accurate and efficient gap closure. To reconstruct the missing component between the two ends of the same insert, the RFfiller meticulously searches for valid overlaps (in repeat regions) and generates transition tables for similar reads, allowing it to make a statistical guess at the missing sequence. Finally, in our experiments, we show that the RFfiller’s gap-closing accuracy is better than that of other publicly available tools when sequence data from various organisms are used. Assembly benchmarks were used to validate RFfiller. Our findings show that RFfiller efficiently fills gaps and that it is especially effective when the gap length is longer. We also show that the RFfiller outperforms other gap closing tools currently on the market.

## Introduction

Rapid DNA sequencing methods have substantially aided research and discoveries in the biological and medical sciences. Basic biological research and a wide range of applications, such as medical diagnostics, biotechnology, forensic biology, virology, and biological systematics, have become more dependent on DNA sequence knowledge (Behjati & Tarpey, 2013). A comparison of healthy and altered DNA sequences can be used to diagnose a variety of disorders, including malignancies, and to describe antibody repertoires and to guide patient treatment (Pekin et al., 2011; Chmielecki & Meyerson, 2014). Having a quick way to sequence DNA enables the identification and cataloging of a greater number of species, in addition to enabling more efficient and more specialized medical care.

Modern DNA sequencing technology has aided in the sequencing of whole DNA sequences or genomes of different types of organisms and species, including the human genome and other DNA sequences of animals, plants, and microbes (Abate et al., 2013; Vega, 2019; Collins, Morgan & Patrinos, 2003). Genome assembly is the bioinformatics process of reassembling a huge number of short DNA sequences to recreate the chromosomes from which the DNA originated (Dida & Yi, 2021). Next-generation sequencing, such as PacBio SMRT sequencing or Nanopore sequencing, is one of the first steps in sequence assembly. PacBio’s SMRT sequencing technology and Oxford’s single-molecule nanopore sequencing technology are two examples of third-generation sequencing technologies that have recently been applied to biological genome sequencing. Third-generation sequencing read lengths are hundreds of times longer than next-generation sequencing and may exceed 10 kbp. Databases such as the European Nucleotide Archive (Leinonen et al., 2010), NCBI Assembly (Coordinators, 2016), and Ensembl Genomes (Hubbard et al., 2009) can be used to store the completed genome assembly. Computer programs typically use single and paired reads to assemble a genome using next-generation sequencing platforms. Depending on the sequencing platform, the length of these reads can range from 20 to 1,000 bp. Paired reads are preferred over single reads because they help link contigs into scaffolds and reveal the size of repetitive regions.

Repetitive sequences, variations, missing data, and errors can all make genome assembly more difficult. The reconstruction of a contiguous genome is made possible by long-read technologies that bridge repetitive regions. Pacific Biosciences(PacBio) and Oxford Nanopore Technologies (ONT) (Reuter, Spacek & Snyder, 2015) are pushing single-molecule real-time (SMRT) and nanopore sequencing for this new generation to increase the number of matches. Assembled draft genomes generally feature many gaps; important biological data, like genes, can be stored within these gaps. For this reason, filling in the gaps may lead to the discovery of unknown information that improves the gene sequence integrity. At present, gaps are filled in five ways: assembly by multiple types of software, use of reference genomes from closely related species, assembly using different types of data, use of polymerase chain reaction amplification at the ends of gaps, and adoption of improved assembly methods based on the de Bruijn graph. This study focuses on assembly using different types of data.

One of the final stages of genome assembly, especially in large genomes, is gap-filling. First, assembly algorithms create contigs, which are contiguous sequences of overlapping sequencing reads. A contig is a contiguous DNA sequence, having no ambiguities or unknown bases, that is denoted by the letter N. Second, scaffolding connects the contigs into longer fragments using specialized sequencing read data. Mate-pair reads were the primary source of scaffolding data until the development of long-read technologies. Mate-pair libraries, also known as jumping libraries, are DNA fragments of sizes ranging from thousands to millions of base pairs that have been size-selected. The fragments’ ends are then sequenced, and the resulting reads link the contigs together. Scaffolds are linked sequences, whereas N-characters represent the unknown sequence between contigs. Long continuous reads, such as those generated by Pacific Biosciences’ RS II or Sequel third-generation sequencing platforms, are frequently used in scaffolding. PacBio is the only sequencing technology that can produce HiFi reads with a precision of > 99.9%, which is comparable to short reads and Sanger sequencing. HiFi reads allow you to detect all types of variants with high precision and recall, including single nucleotide and structural variants, as well as phase haplotypes, even in difficult-to-sequence portions of the genome. (Accurate circular consensus long-read sequencing improves variant detection in and the assembly of a human genome.) The scaffolding process connects and arranges the contigs, although the result typically comprises many unknown sequences. Gaps are the unidentified sequences. Finally, with or without further sequencing data, the gap-filling stage seeks to resolve these unknown sequences. Even after the gap-filling procedure, many published genomes still contain significant gaps.

Hence, several tools have been designed to close the gapped regions using sequence reads, including TGS-GapCloser (Xu et al., 2020), SOAPdenovo GapCloser (Luo et al., 2012), and Sealer (Paulino et al., 2015). TGS-GapCloser closes gaps in large genomes more efficiently and accurately than other gap-closing tools by using low-coverage, error-prone long reads (Xu et al., 2020). TGS-GapCloser takes any type of TGS long read or other pre-assembled contig as input and fills gaps in a draft assembly in four steps: gap detection in the draft assembly, candidate acquisition from long-read alignments against gaps, base-level error correction of alternative sub-long reads, and gap closure using the highest-scoring error-corrected candidates for each gap or linkage of neighboring scaftigs with overlaps.

Using the ample pair relationships of short reads, the GapCloser (Luo et al., 2012) was designed to close the gaps discovered by SOAPdenovo or other assemblers during the scaffolding process. Scaffolds were built in the original SOAPdenovo (Li et al., 2010) using PE reads, beginning with small insert sizes (200 bp) and progressing iteratively to large insert sizes (10 kbp), which resulted in lower scaffold quality and shorter scaffold length. SOAPdenovo improved the original GapCloser module in SOAPdenovo2, which iteratively assembled sequences in the gaps to fill large gaps. The previous GapCloser only considered the reads that could be aligned within the current iterative cycle at each iteration. SOAPdenovo developed a new approach for SOAPdenovo2 that considered all reads aligned during previous cycles, which allowed for better resolution of conflicting bases and thus improved gap closure accuracy.

Lastly, Sealer (Paulino et al., 2015) is an automated finishing application that closes gaps in draft assemblies, including those of very large genomes, using a succinct Bloom filter representation of the de Bruijn graph. The sealer has three distinct functions. First, regions with Ns (from an input scaffold file) are identified, and the nucleotides flanking each gap are extracted. To connect the flanking sequences, the Konnector utility (Vandervalk et al., 2014) is used with a variety of *K*-mer lengths. Finally, successfully connected sequences are inserted into the gaps of the original scaffolds, resulting in the generation of a new gap-filled scaffold file.

In this article, we introduce RFfiller, a hybrid genome gap filler that, unlike most gap fillers, avoids the consensus procedure entirely, does not follow the overlap layout consensus or de Bruijn graph paradigms, and uses long reads in the early stages of gap filling to further analyze the gap regions. Standard assembly benchmarks were used to validate RFfiller. Our findings show that RFfiller efficiently fills gaps, and that it is especially effective when the gap length is long. We also show that RFfiller outperforms other gap-closing tools that are currently on the market.

## Methods

**Figure 1:**
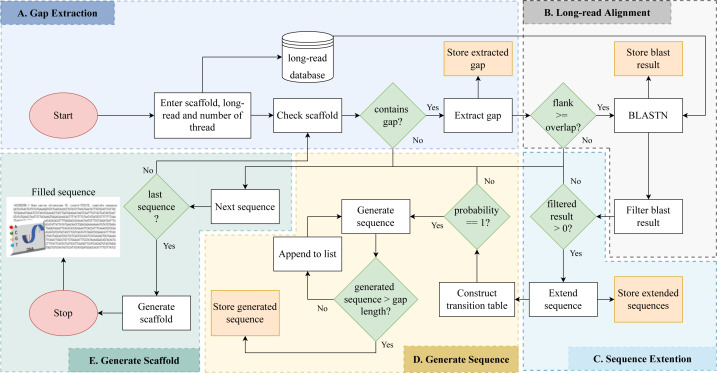
Detailed flowchart of RFfiller. (A) The input scaffold will be checked for gaps. Gaps will be extracted and stored in a separate file if they exist. (B) Once the gaps are stored, the extracted gaps and long reads will be used to perform long read alignment. (C) The aligned sequence will be extended to cover the entire region of the gap they represent based on the results of the long read alignment. (D) After analyzing the extended sequence pattern, a transition table will be generated. The transition table is used to generate missing gap sequences based on the pattern extracted from the extended sequences using Markov Chains. (E) A new scaffold will be generated with the newly generated sequence replacing the missing sequences.

Gap detection, long read alignment, read extension, transition table construction, Markov chain construction, and gap-filling are the five stages of RFfiller. This section summarizes the procedures that comprise the RFfiller algorithm. Figure 1 illustrates the process in detail.

### Gap detection

**Figure 2:**
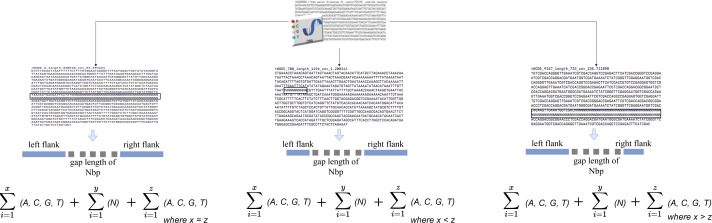
Gap extraction from an input file. Where *x, y* and *z* represents sequence length. N represents gaps or unknown sequence.

The algorithm iterates across the scaffold in this stage to look for gaps or an unknown sequence (*i.e.*, the letter N). When a gap is discovered, the surrounding sequences are retrieved based on an overlap threshold. There are three scenarios involved in the extraction, as illustrated in Fig. 2. The first scenario determines if there are enough neighboring sequences to extract from both ends of a gap, which results in an equal number of overlapping sequences surrounding the gap. The second scenario determines whether there are sufficient sequences on the gap’s left side, which results in the extraction of as many sequences as possible from the left side. The third scenario is identical to the second but for the right side of the gap. The second and third scenarios also evaluate whether both ends of the gap contain an adequate sequence for extraction. The remaining sequence from both sides is retrieved in this case.

When a gap is discovered and its neighboring sequence is extracted, it is stored in a file with the ID of the sequence in which the gap was discovered.

### Long-read alignment on the gap region

The gap files that are stored are the focus of this stage. The gap files goes through each saved file one by one, searching for a matching sequence by using a long-read alignment on the gap region.

Long-read alignment can be accomplished using a variety of bioinformatics methods. The Basic Local Alignment Search Tool (BLAST) (McGinnis & Madden, 2004) is a well-known aligner. BLAST searches for sequences that share local similarities. Comparing nucleotide or protein sequences to databases and calculating the statistical significance of matches are the primary functions of this program. With the use of BLAST, it is possible to infer functional and evolutionary links between sequences, as well as to identify members of gene families.

BLAST uses a heuristic method to find short matches between two sequences, so it does not consider the entire sequence space when doing its work. The long-read and the gap regions are input sequences. To find sequence information quickly, we create an index of the long-read. Then, we create a BLAST database to search against the long-read sequences. The long read is compared with the gap region, and any matches found are recorded. BLAST tries to attempts to begin local alignments from the initial matches after the first match.

To reduce the number of similar reads, we limit the length of the gap region; if all of the gap regions were allowed to align with the long read, the likelihood of having thousands of similar matches for a specific location would be high. We also set a limit on the percentage of aligned sequences that are identical. Percentage identity is a number that expresses how similar the query and target sequences are (how many characters in each sequence are indistinguishable). The more significant the match, the higher the percentage identity. Based on this premise, we set the percentage identity to 97% to fill the gap with a proper sequence, and any sequence having a match of 98 percent or greater is considered a high-quality match. This later helps the Markov chain to infer accurate reads. The more accurate the reads generated by the blast, the more accurately the Markov chain infers.

The length and location of the match are also restricted. Even if a short-length match were found far away from the gap, it would not be considered a match, regardless of its percentage identity. Due to its alignment length and location, such a sequence does not provide valuable information about the gap; if the aligned sequence were similar to the gap and gap region, it would have longer matches that span the entire gap region or at least one end. Any aligned sequence that fails to meet this constraint is removed from the alignment. The alignment statistics generated by BLAST are stored in a separate directory for use after the alignment process is completed.

### Read extension on aligned sequences

At this stage, we go through each gap’s stored filtered alignment files one by one. Our confidence in the sequence alignment allows us to filter the alignment so that it only contains the information we require (*i.e.*, subject and query id, subject and query start, and subject and query end).

**Figure 3:**
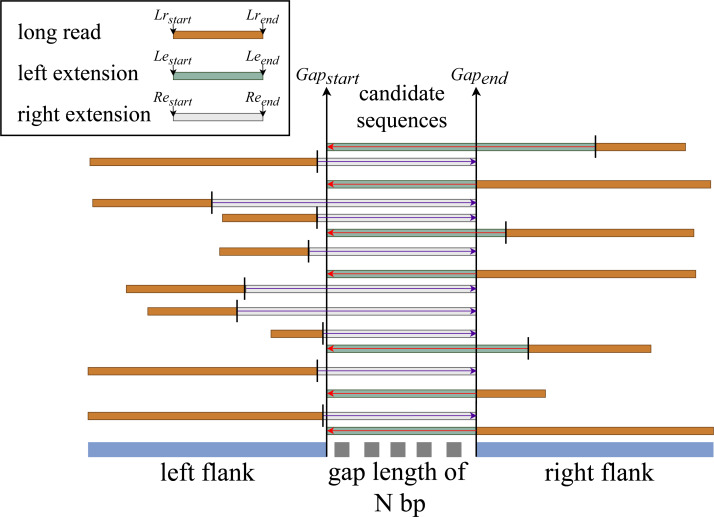
General flow of right and left sequence extension on the aligned reads.

We hypothesize that if there is an aligned sequence with sufficient information that meets the constraint set at stage two, then by extending that sequence within the gap’s range, we have a good chance of filling the gap. We then perform the left and right extension procedures for all match sequences based on this hypothesis, as illustrated in Fig. 3.

Before the extension procedure, we perform a sequence directionality check on the alignment statistics file. Sequence directionality (Lodish et al., 2008) is the end-to-end chemical orientation of a single strand of nucleic acid. In a single strand of DNA, the chemistry convention of naming carbon atoms in the nucleotide pentose-sugar-ring means that there will be a 5′-end, which frequently contains a phosphate group that is attached to the 5′  carbon of the ribose ring, and a 3′-end, which is typically unmodified from the ribose -OH substituent. In a DNA double helix, the strands run in opposite directions to permit base pairing between them. A gene is read in the 3′  to 5′  direction (forward sequence), and so its REVERSE complementary strand is read in the 5′  to 3′  direction (reverse sequence). Based on sequence directionality, we split the filtered alignment into the forward and reverse sequence directions. We do this by checking if the query start is larger than the query end. If the query start is greater, the directionality of the sequence is reversed.

After we determine the sequence directionality, we pass it on to the left and right extension procedures.

#### Left extension

This procedure is only applicable to sequences that are aligned with the gap’s right end (right flank). It extends the aligned sequence from the aligned sequence’s start index to the gap’s start index.

This procedure first determines whether the given sequence is moving in the correct direction. If the sequence is moving in the forward direction, subject start—(gap length + (query start - gap end))—is used to create a new start index. RFfiller then checks if the newly generated start index is contained within the subject sequence’s length. We must check if the extension index is within the sequence because we are extracting the gap-filling sequence from the subject sequence (long-read alignment). If the new start index is not within the range of the subject length, it means that the extended read resides at the beginning of the subject length. If this is the case, we set the new start index to zero. After confirming that the index can be used, the new extension end index is created by simply adding the gap length to the newly created start index.

If the sequence directionality is reversed, we check if the newly generated end index is less than the sequence length. Because the query start is greater than the query end, the extension of the gap length usually extends the limit of the subject sequence. In this case, we set the new end index to the subject length, and the new start index becomes the difference between the new start index and the gap length. Now that we have the final left extended sequence index, we write the subject id, new start index, new end index, and its directionality to a file.

#### Right extension

This procedure is almost the same as the left extension but works on sequences that are aligned to the left end of the gap (left flank). It extends the aligned sequence starting from the aligned sequence end index to the end index of the gap. The rest of the process is the same except when it computes the new start index, it uses subject end + (gap start - query end) for forward sequence directionality and subject end - (gap length + (query start - query end)) for reverse sequence directionality.

The Browser Extensible Data (BED) file format is used to save the files. The BED (Quinlan & Hall, 2010) format is a text file format for storing genomic regions as coordinates and annotations. The information is organized into columns that are separated by spaces or tabs. One advantage of this format is that it compares genomes using coordinates rather than nucleotide sequences, which improves power and computation time.

### Construction of a transition table

At this stage, we access the stored BED files. The BED files contain candidate sequence indexes to fill all of the scaffold’s gaps. We use BEDTools, a specialized tool for parsing BED files, to convert the index to a sequence. We pass the BED files and the long-read sequence as input parameters. The BEDTools look for indexes in the long-read sequence and then check the sequence’s directionality. If the sequence directionality is forward, the tool searches the index on the long-read sequence directly and outputs the found sequence. However, if the directionality is reversed, it applies the reverse complement to the discovered sequence by searching the index.

We now have enough information about the sequences to fill the gap after converting the indexes into reads. RFfiller generates a transition table for each of the candidate sequences for further analysis. The transition table is a table that shows how the transition function works. It takes two inputs (a state and a symbol) and outputs a state (the “next state”). A transition table is represented by columns corresponding to input symbols.

**Figure 4:**
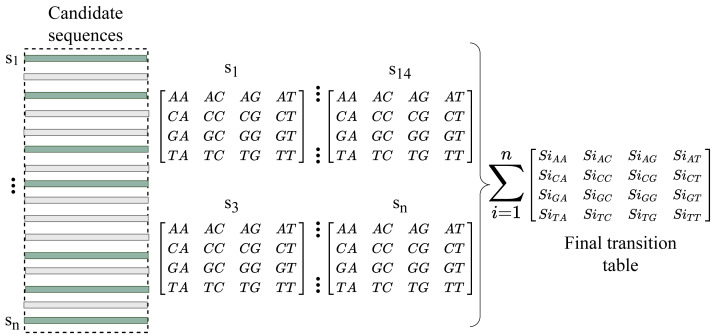
Construction of transition table from candidate sequences.

RFfiller builds a transition table by counting the frequency of each nucleotide in the candidate sequence. To calculate the frequency, we must first convert the sequences into numerical representations: *A* = 0, *C* = 1, *G* = 2, and *T* = 3. We then count the frequency of each nucleotide pair (how many times a nucleotide follows another nucleotide). We perform this operation for all the candidate sequences, as illustrated in Fig. 4.

After constructing the frequency table, we use probability to convert it to a transition table. In a frequency table, the total sum of a row is used as a divider for the columns within the row. To prevent a division-by-zero error, the sum should always be greater than zero. In some cases, however, we allow the sum to be zero; when a candidate sequence is only one nucleotide long. In this case, we set the rest of the rows to zero to maintain the integrity of the transition table dimension. The final transition matrix is indexed with A, C, G, and T to complete this step.

### Markov chain

**Figure 5:**
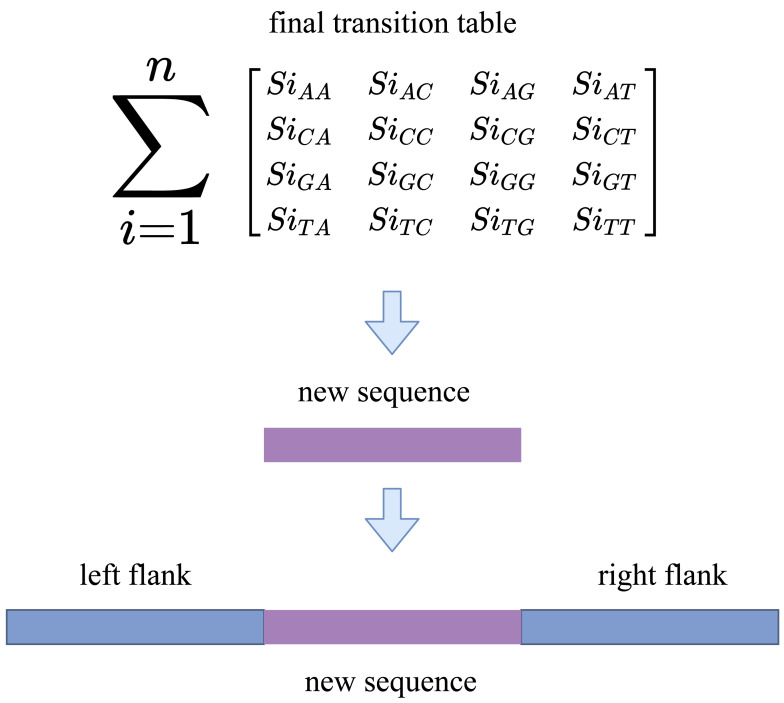
Gap filling using Markov chain with 16 candidate sequences.

This stage takes the transition matrix, gap length, and last character before the gap stars (last character) as input parameters. RFfiller generates the final sequence based on the transition matrix, as illustrated in Fig. 5. It first converts the last character to uppercase. This is because some scaffolds are of mixed-case nucleotides (lower- and uppercase), and it disrupts the transition matrix because it is indexed on the uppercase of the nucleotides. It then tries to guess, given the last character, only one nucleotide. This is one of the most crucial steps in this stage. Providing the last character base assures the sequence to be generated is following a pattern from the gap region. RFfiller also guarantees that the Markov chain does not start at a random location. This means that there will always be prior knowledge of the gap before sequences that fill it are generated.

 The Markov chain generates the same number of nucleotides as the number of gaps present. We repeat this stage 5–10 times for a single gap because it is a statistical procedure. Now that we have multiple sequences, the next step is to calculate the probability of each newly generated sequence and to select the one with the highest probability. The transition matrix is used to calculate the sequence probability. It checks the probability of the first nucleotide to its preceding nucleotide and records the transition cost, given one of the final sequences. There are n-1 transition costs for an n-length sequence. The sequence rank is determined by the average transition cost of a sequence.

### Gap filling

The algorithm creates an output file similar to the scaffold sequence now that we have gathered the sequences to fill the gap. If there are gaps in the scaffold, the algorithm searches for the sequence ID within the newly generated sequences before creating the output file. If the sequence ID exists, the newly generated sequence fills the gap. After that, it is saved to the output file. RFfiller does not affect the rest of the sequence, even if a newly created sequence is not found. It only replaces unknown sequences (labeled by the letter ‘N’).

## Results

### RFfiller algorithm

Long reads are used to generate a sequence based on a statistical outcome, followed by sequence assembly using a Markov chain-based gap-filling technique. By providing sufficient information about the gap region, RFfiller can forecast the next nucleotide given the current state using the Markov chain concept.

The RFfiller algorithm fills gaps using both a scaffold and a long-read sequence from the same organism. When the scaffold contains unknown sequences or gaps, as in this case, the algorithm is efficient. The algorithm attempts to identify gaps by examining the neighborhood sequence in which they occur. Due to the presence of duplicates in the gap region, it is necessary to inspect the gap region itself. In the gap region, a pattern is determined. This pattern serves as a starting point for evaluating the newly discovered sequence.

**Figure 6:**
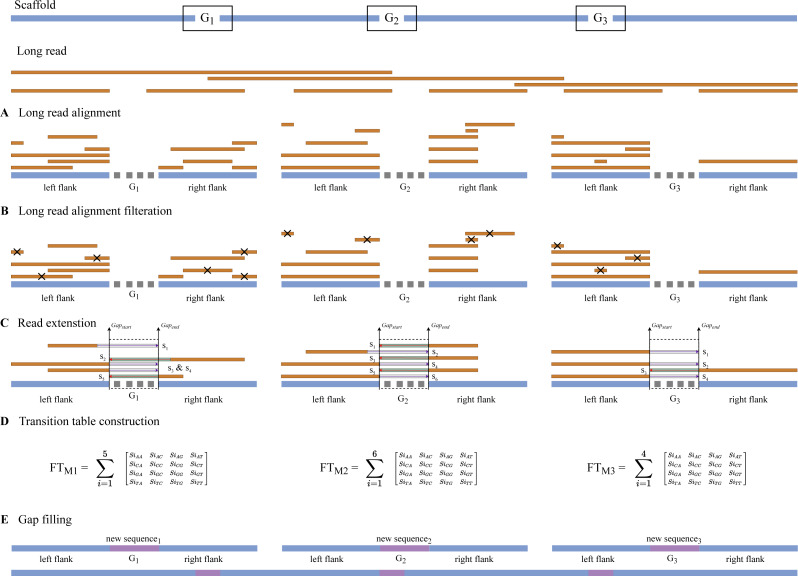
The RFfiller algorithm. RFfiller workflow consists of first detecting and extracting gaps from the input scaffold. (A) The long-read alignment is used to find sequences that are similar to the gap region. (B) Once similar reads are found, based on their percentage identity and location, similar reads are filtered out. (C) The remaining reads will be extended based on their directionality to cover the gap length. These reads are called candidate reads. (D) Based on the candidate reads, a pattern will be determined by counting the frequency of the nucleotides by constructing a transition table. The aggregated result of each candidate reads transition table will generate the final transition table, which will be applied to the Markov chain. (E) The output of the Markov chain will be used to fill the gaps.

The long read may generate read fragments that are identical to those found in the gap region, indicating that the long read may contain the same sequence as the gap region. When the algorithm discovers similar reads, it replaces the unknown sequence with read fragments from those candidates using a Markov chain. The output of the Markov chains is used to close the gap. As illustrated in Fig. 6, RFfiller consists of five stages: gap detection, long-read alignment, read extension, transition table construction, Markov chain construction, and gap filling. Each stage is discussed in detail in the Methods section.

### Experiment

To assess accuracy, assemblies were compared with finished sequences derived from independent sequencing experiments. Assemblies were created using popular assembly programs with default parameters as a way to compare the naively generated assemblies. Some gap fillers used short reads while others used long reads. We intended to compare how well a gap filler fills a gap given the same input. In total, there were 36 assemblies from six assembly programs and six datasets. Using the output of the assemblers, we performed 144 gap-filling experiments using four gap-filling algorithms. All experiments were performed on the same server (two Intel Xeon E5-2695 v4 processors with a memory limit of 128 GB). All the parameters used to conduct this experiment can be found in the supplementary material. To keep the article concise, we have chosen to present only the key metrics from each gap filler result in the subsequent evaluations.

#### Dataset

In this experiment, we used three bacteria, a mammal, a plant, and a fungus. This included *Arabidopsis thaliana*, *Bacillus cereus*, *Escherichia coli*, *Homo sapiens*, *Saccharomyces cerevisiae*, and *Staphylococcus aureus* WGS data. All the species we incorporated had previously been sequenced and completed using one of the assemblers mentioned in the manuscript to a very high level. The correctness of each assembler was rigorously evaluated and compared using the reference genome.

The six genomes represent a wide range of genome sizes, from bacteria to the human genome. Because some of the assemblers would require several weeks to assemble the entire genome, and others would eventually fail, a sample having a wide range of read length and coverage was chosen. Table 1 summarizes the details of the reads used for the experiment.

**Table 1:** Dataset information.

**Dataset**	**Read type**	**Technology**	**Accession number**	**Refseq**	**# bases**	**Coverage**
***Arabidopsis thaliana***	Short	Illumina	ERR3485043	GCF_000001735.4	304.3M	2.3
	Long	PacBio	ERR3415827		1.9G	8.7
***Bacillus cereus***	Short	Illumina	ERR3338758	GCF_000007825.1	443.6M	3.0
	Long	PacBio	SRR9641613		1.2G	25.6
***Escherichia coli***	Short	Illumina	SRR12573761	GCF_000005845.2	326.7M	62.9
	Long	PacBio	SRR10538960		3.3G	488.9
***Homo sapiens***	Short	Illumina	SRR005721	GCF_000001405.39	860.9M	N/A
	Long	PacBio	SRR13684281		6.8G	2.3
***Saccharomyces cerevisiae***	Short	Illumina	SRR12596359	GCF_000146045.2	3.0G	225.0
	Long	PacBio	ERR4467305		5.3G	288.7
***Staphylococcus aureus***	Short	Illumina	SRR12560295	GCF_000013425.1	480.7M	167.8
	Long	PacBio	SRR10807892		2.5G	715.5

#### Assemblers

We chose six *de novo* assemblers for this experiment. Of the six, three are short-read assemblers (ABySS, SPAdes, and SOAPdenovo2), and the remaining three are long-read assemblers (A5-MiSeq, Flye, and SGA).

SPAdes (Bankevich et al., 2012), SOAPdenovo2 (Luo et al., 2012), and ABySS (Simpson et al., 2009) are short-read assemblers. SPAdes is a single-cell, standard A-Bruijn assembler (Pevzner, Tang & Tesler, 2004). It alters the graph topology, coverage, and length of sequences rather than the sequences themselves. It uses only *K*-mers to build the de Bruijn graph. The final stage restores the DNA consensus sequence. For the large genome, SOAPdenovo2 is designed to solve a greater number of repeated contiguous areas, which increases scaffolding coverage and length. Lastly, ABySS (Assembly By Short Sequences) is a distributed representation of a de Bruijn graph, which allows the assembly algorithm to be parallelized across a network of commodity computers. The ABySS algorithm is split into two parts. The sequence reads are first used to generate all possible substrings of length k (known as *K*-mers). Read errors are then removed from the *K*-mer dataset, and the initial contigs are created. Mate-pair information is used in the second stage to extend contigs by resolving ambiguities in the contig overlaps.

The long-read assemblers are A5-MiSeq (Coil, Jospin & Darling, 2015), Flye (Kolmogorov et al., 2019), and SGA (Simpson & Durbin, 2012). SGA is an assembler based on FM index (Ferragina & Manzini, 2005; Burrows & Wheeler, 1994), memory-efficient data structures, and assembly algorithms. Preprocessing the sequence reads to filter or trim the reads is the first step in the SGA pipeline. The FM index is built from the filtered set of reads, and base-calling errors are detected and corrected using *K*-mer. Corrected reads are re-indexed, duplicate sequences are discarded, and a string graph is generated. Scaffolds are built from the string graph if paired-end or mate-pair data are available. Each step in the A5-MiSeq pipeline is described below. The specifics are as follows: (I) Trimmomatic (Lohse et al., 2012) removes adapters and low-quality sequences. This is followed by read error correction using the SGA *K*-mer. (II) IDBA-UD (Peng et al., 2012) assembles paired and unpaired reads. (III) Any large insert library can scaffold contigs with permissive parameters. Misassembled contigs and scaffolds are broken. Following this, a final round of scaffolding repairs any previously broken continuity. Finally, Flye, a long-read assembly algorithm that builds an accurate repeat graph from disjointigs, generates disjointigs that represent disjointed genome segment concatenations. Error-prone disjointigs are then joined together. Assembly graphs are built using the resulting string read, which untangles the graph and fixes bridged repeats. Later, the repeat graph addresses unbridged repeats and outputs precise contigs formed by the paths in the graph.

Except for the human genome, we ran the assembler using the default parameters to allow comparisons between assemblies produced by different assemblers. We fine-tuned the parameters of each assembler because the default parameters were inadequate to enable the assembler to run on the human genome. All of the assembler’s output contains an unknown sequence. Using their output, we tested the gap-filling tools.

#### Validation

To determine the best assembly for each gap-filling tool, QUAST QUAST (Gurevich et al., 2013) was used to determine length statistics for the assembly, such as total length and NG50, as well as alignment to the reference, including NGA50, mismatch, number of gaps, total aligned length, and misassemblies.

• **NG50** is the length for which the collection of all contigs of that length or longer covers at least half the reference genome.
• **NGA50** is an NG50 corrected assembly error.
• **Mismatch** is the number of mismatches in all aligned bases.
• **The number of gaps** is equal to the total number of uncalled bases (N’s) in the assembly.
• **Misassemblies** is the number of positions in the assembled contigs where the left- flanking sequence is more than 1 kbp away from the right-flanking sequence on the reference (relocation), or where flanking sequences are on different strands (inversion), or different chromosomes (translocation).

### Evaluation

This section intends to demonstrate which gap-filling algorithm generates the fewest mismatches and misassemblies, as well as the greatest number of NGA50 and total aligned length, on the filled gap sequence. Additionally, gap-filling algorithms are evaluated in terms of different flanks and gap length, including an equal number of flanks, a single-sided flank, a shorter flank, and a gap length of 1 bp. Different flanks and gap lengths in the same dataset may fall into different categories. According to our analysis of the sequences, those sequences having a large number of 1 bp gaps fell into the 1 bp gap-length category. The same was true for the remaining categories.

#### Equal number of flanks

**Figure 7:**
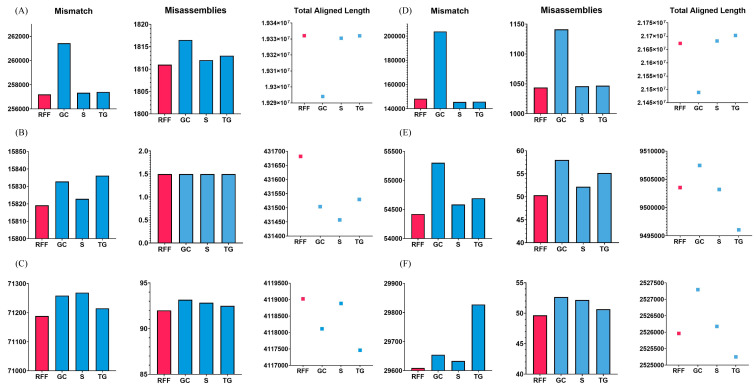
Assembly statistics. Misassemblies, mismatch and total aligned length of gap fillers on (A) *Arabidopsis thaliana*, (B) *Bacillus cereus*, (C) *Escherichia coli*, (D) *Homo sapiens*, (E) *Saccharomyces cerevisiae*, and (F) *Staphylococcus aureus* dataset. RFF represents RFfiller, GC represents GapCloser, S represents sealer, and TG represents TGS-GapCloser. The *y*-axis represents basepairs. The *x*-axis represents gap fillers.

To validate this case, the *Arabidopsis thaliana*, *Bacillus cereus*, and *Staphylococcus aureus* datasets were used. RFfiller generated the fewest misassemblies and mismatches. Additionally, it generated the greatest number of total aligned lengths compared with the other gap fillers, as illustrated in Fig. 7. These datasets contain an equal number of flanking sequences. RFfiller took advantage of this to amass sufficient overlap information to construct a well-defined transition table. The precise unknown sequences were then generated using the well-defined transition table. On the three datasets used to validate this concept, RFfiller outperformed the other gap-filling algorithms for an equal number of flanks. Table 2 summarizes the output of the gap fillers.

**Table 2:** Difference in assembly statistics of all gap fillers and *de novo* assemblers across all datasets.

**Assemblers**	**Gap filling algorithms**	**Arabidopsis thaliana**					**Bacillus cereus**					**Escherichia coli**				
		**# gaps**	**Misassembly**	**Mismatch**	**TAL**	**NGA50**	**# gaps**	**Misassembly**	**Mismatch**	**TAL**	**NGA50**	**# gaps**	**Misassembly**	**Mismatch**	**TAL**	**NGA50**
**A5-MiSeq**	**RFfiller**	0	***10***	***1009***	***−17***	–	0	0	***20***	***−160***	–	0	0	***4***	***−7***	0
	**GapCloser**	**63610**	−24	−24172	229264	–	***799***	0	−3	−108	–	***558***	0	−127	0	0
	**Sealer**	220	2	332	12974	–	8	0	−6	−141	–	1	0	1	22	0
	**TGS-GapCloser**	0	0	0	0	–	0	0	0	0	–	0	0	0	0	0
**ABySS**	**RFfiller**	0	0	0	0	–	303	0	***19***	***−516***	–	288	***2***	***26***	***−578***	328
	**GapCloser**	0	0	0	0	–	***855***	0	−16	−409	–	***952***	−2	−10	−372	328
	**Sealer**	0	0	0	0	–	789	0	−6	−194	–	718	−1	−19	−556	328
	**TGS-GapCloser**	0	0	0	0	–	100	0	0	0	–	202	0	−48	−1924	***0***
**FLYE**	**RFfiller**	0	0	0	0	0	0	5	0	0	–	0	0	0	0	0
	**GapCloser**	0	0	0	0	0	0	0	0	0	–	0	0	0	0	0
	**Sealer**	0	0	0	0	0	0	5	0	0	–	0	0	0	0	0
	**TGS-GapCloser**	0	0	0	0	0	0	0	0	0	–	0	0	0	0	0
**SGA**	**RFfiller**	0	0	0	0	–	50	0	***8***	***−1060***	–	0	0	0	0	1464
	**GapCloser**	0	0	0	0	–	***864***	0	−2	61	–	0	0	0	0	1464
	**Sealer**	0	0	0	0	–	0	0	0	0	–	0	0	0	0	1464
	**TGS-GapCloser**	0	0	0	0	–	175	0	−55	−1032	–	0	0	0	0	1464
**SOAPdenovo2**	**RFfiller**	763	2	−64	−188	–	786	0	0	−163	–	1343	***1***	***14***	***−11545***	***−51***
	**GapCloser**	***2938***	3	***−16***	−1637	–	***5030***	0	−16	***−713***	–	***4103***	−2	−234	−6465	40
	**Sealer**	2880	***4***	−24	***−4577***	–	4857	0	***32***	−408	–	3807	−1	−415	−11068	−29
	**TGS-GapCloser**	897	0	−67	−628	–	87	0	0	7	–	393	0	−58	−1355	−29
**SPAdes**	**RFfiller**	***200***	0	***199***	***−805***	–	100	0	***6***	***−45***	–	200	0	***0***	***−300***	4722
	**GapCloser**	80	0	−39	−258	–	***695***	0	2	297	–	***447***	0	−7	−134	4722
	**Sealer**	0	0	0	0	–	600	0	5	153	–	300	0	−6	6	4722
	**TGS-GapCloser**	0	0	0	0	–	0	0	0	0	–	200	0	−9	212	4722
		**Homo sapiens**					**Saccharomyces cerevisiae**					**Staphylococcus aureus**				
**A5-MiSeq**	**RFfiller**	118	***21***	***2007***	***38***	–	−5647	2	***−8***	***−1000***	0	6097	0	***−9990***	***0***	0
	**GapCloser**	***697827***	−562	−347997	1186684	–	***24***	***3***	−3885	−90	0	***6137***	0	−10012	***0***	0
	**Sealer**	704	12	589	69560	–	−5965	1	−80	−567	0	6097	0	−9998	265	0
	**TGS-GapCloser**	187	0	−6	102	–	−5729	1	−42	−859	0	6097	0	−10000	***0***	0
**ABySS**	**RFfiller**	0	0	0	0	–	−5472	***4***	***2096***	***−16125***	***−100***	4917	−25	***−300***	***−1200***	−16603
	**GapCloser**	0	0	0	0	–	***199***	−3	−1781	−15215	−1	5068	−25	−322	−130	−16603
	**Sealer**	0	0	0	0	–	−5790	0	2024	−15692	−81	***5075***	−25	−308	0	−16603
	**TGS-GapCloser**	0	0	0	0	–	−5554	−3	2062	−15984	3147	5022	−25	−310	−401	−16603
**FLYE**	**RFfiller**	0	0	0	0	–	−300	0	0	***0***	–	300	0	0	0	0
	**GapCloser**	0	0	0	0	–	***−200***	0	***13***	262	–	300	0	0	0	0
	**Sealer**	0	0	0	0	–	−300	0	0	***0***	–	300	0	0	0	0
	**TGS-GapCloser**	0	0	0	0	–	−300	0	0	***0***	–	300	0	0	0	0
**SGA**	**RFfiller**	0	0	0	0	–	−2100	***8***	***−83***	−20018	***0***	10926	***4***	***104***	***−3911***	***−603***
	**GapCloser**	0	0	0	0	–	***4737***	−13	−511	−11565	***0***	***14173***	−3	−63	−3771	−25
	**Sealer**	0	0	0	0	–	−3803	2	−229	−3706	3297	9268	0	0	0	0
	**TGS-GapCloser**	0	0	0	0	–	−66	−9	−411	−2887	6978	12401	1	−917	5649	−537
**SOAPdenovo2**	**RFfiller**	***171396***	−2	−17853	123776	–	−22310	−7	***−477***	−41611	1213	39137	−2	−202	−894	0
	**GapCloser**	164706	***14***	−1209	43796	–	***2992***	−23	−1495	***−89720***	2098	***41044***	−10	−228	***−10522***	178
	**Sealer**	0	0	***0***	0	–	−7817	***−6***	−1320	−75616	***−820***	40348	−10	***−178***	−7659	178
	**TGS-GapCloser**	2521	1	−62	***−4041***	–	−32770	−10	−1455	−32457	694	38116	***0***	−422	−7659	***−71***
**SPAdes**	**RFfiller**	***1534***	***6***	***34***	−209	–	−4639	***3***	−136	***−10592***	0	7439	0	***9***	***−1069***	0
	**GapCloser**	1153	−12	−2	−1393	–	−658	0	−115	−3607	0	***7733***	0	−46	−638	0
	**Sealer**	0	0	0	0	–	***−51***	2	***−66***	1739	0	7538	0	−45	−965	0
	**TGS-GapCloser**	190	***6***	***−753***	***−49977***	–	−439	2	−168	2094	0	7339	0	−17	−379	0

**Notes.**

Values in italicized bold indicate the best result in each category.

The full results of gap fillers and *de novo* assembly statistics on each dataset can be found in the Supplementary Material.

#### One flank shorter

When RFfiller was executed, few misassemblies and mismatches were produced. Additionally, when compared with the other gap fillers, it produced the greatest number of total aligned lengths, as illustrated in Fig. 7. The *Escherichia coli* overlap data aided in verifying that one the flank was shorter than the other flank. Although RFfiller extracted less information than the algorithms utilized on the *Arabidopsis thaliana*, *Bacillus cereus*, and *Staphylococcus aureus* datasets, the overlap information obtained from both flank ends was adequate to construct an appropriate transition table. Despite this, RFfiller outperformed the other gap fillers in terms of misassemblies, mismatch, and total aligned length on the scaffold constructed by all de novo assemblers. Table 2 summarizes the gap filler’s output.

#### Single-sided flanks

As illustrated in Fig. 7, RFfiller had the fewest misassemblies and mismatches. RFfiller was evaluated against single-sided flanks on the *Saccharomyces cerevisiae* dataset in terms of creating the longest aligned length achievable. *Saccharomyces cerevisiae* data assisted in confirming the condition characterized by the presence of only one side flank. Only one flank was used to retrieve the overlap information. Due to RFfiller inability to discern patterns in such data, information from a single flank is insufficient to accurately narrow a gap. When this occurs, RFfiller fills only a portion of the gap and leaves the remaining portion unchanged. Despite the absence of overlap information, RFfiller outperformed other gap fillers in terms of misassemblies and mismatches. Table 2 summarizes the result of the gap fillers on the *Saccharomyces cerevisiae* dataset.

#### Gap length of 1 bp

RFfiller produced the fewest misassemblies, as seen in Fig. 7. The *Homo sapiens* dataset was used to confirm that the gap length was limited to a single character (1 bp gap length) in some cases. RFfiller gathered overlap information from the flanks in the circumstances described above. Because a gap is 1 bp long, the algorithms’ candidate sequences were all limited to generating 1 bp sequences. A pattern could not be generated from these candidate sequences, resulting in an incorrect transition table. When the algorithm attempted to generate candidate sequences, it looked for patterns within the candidate sequences in order to generate a transition table. A Markov chain would be able to infer the missing sequences based on the transition table and the pattern detected in the candidate sequences. If no pattern was found, no transition table was built (*i.e.*, all the rows and columns are set to 0), and no inference was made. There had to be at least 2 bp for a pattern to be detected. Such a gap was labeled as unfillable by RFfiller. This was especially apparent on the SOAPdenovo scaffold, but the suggested approach also functioned well on scaffolds that were created by other *de novo* assemblers. The results of the gap fillers on the *Homo sapiens* dataset are summarized in Table 2.

### Performance

**Figure 8:**
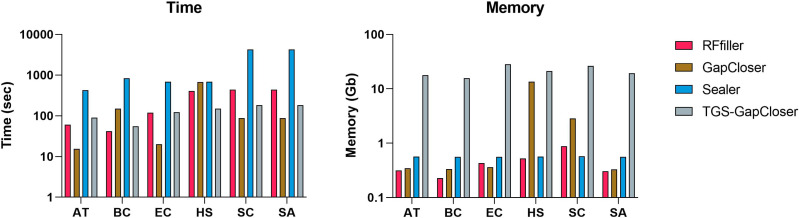
Average time and memory of gap fillers. The *x*-axis represents datasets. AT represents *Arabidopsis thaliana*, BC represents *Bacillus cereus*, EC represents *Escherichia coli*, HS represents *Homo sapiens*, SC represents *Saccharomyces cerevisiae*, and SA represents *Staphylococcus aureus* dataset.

We compared RFfiller with the three gap fillers in terms of time and memory consumption. In comparison with the other gap fillers, RFfiller only utilizes reads that are in the neighboring region of the gap sequences, thus RFfiller outperformed other gap fillers in terms of memory usage. For comparing in terms of time, gap filling is dependent on the gap filler’s objective. For example, if a gap filler’s objective is to fill all gaps within a scaffold at the expense of accuracy, the gap filler will run faster. However, if its objective is accuracy rather than speed, it will make extensive use of memory and time. RFfiller’s objective is to find accurate overlapping sequences to tackle mismatches and misassemblies. It achieves its object at the expense of time. Nonetheless, by extracting sufficient overlapping information from gap regions, a highly accurate read can be achieved within a short period of time (Fig. 8). For this reason, we compared gap fillers based on their ability to fill gaps with fewer mismatches and misassemblies relative to the original sequence. RFfiller was the most resource-efficient algorithm of all the gap fillers. The average result of the comparison is illustrated in Fig. 8.

## Conclusion

Despite the recent advances in next-generation sequencing technologies, published draft assemblies of small and large genomes still contain unknown sequences. We demonstrated in this paper that RFfiller, which is based on a Markov chain, produces high-quality results on most datasets tested while requiring only moderate computational resources. Although RFfiller produces the shortest erroneous sequence length, we cannot generalize about the robustness of our problem formulation because gaps were left unfilled. Although achieving 100% completion without at least some computer-assisted manual finishing and labor-intensive PCR work is improbable, each tool performs differently in terms of misassemblies and the number or length of gaps closed, and this emphasizes the problem difficulty. We demonstrated how long and short reads can be used in the gap-filling algorithms to achieve in a near-complete genome. Additionally, we discovered that patterns exist within a gap region. Using long reads and gap region analysis, we were able to statistically guess missing sequences.

After scaffolding the draft genomes, RFfiller assists in filling long gaps and solving the gap-filling problem. Because no other application employs probability to solve this type of problem, RFfiller is novel. We demonstrated this by comparing the results of our tool to those of Sealer, TGS-GapCloser, and GapCloser.

Sealer and GapCloser utilized short reads to close the gap. In our findings, we discovered that both Sealer and GapCloser reconstructed the scaffold in the absence of a gap. In this case, their assembly statistic was not better than the provided scaffold. GapCloser was somewhat greedy when closing gaps. It was more concerned with filling in the gaps than with accuracy. Due to its lack of focus on accuracy, it excelled at time and memory efficiency.

TGS-GapCloser and RFfiller closed the gaps using long reads. Their result was extremely similar, but TGS-GapCloser generated significantly more mismatches than did RFfiller. Even when fewer mismatches and misassemblies were generated, the total aligned length and fully aligned contigs were shorter than the provided scaffold and RFfiller output. Similarly, similar scenarios were observed with Sealer. Concerning resource consumption, TGS-GapCloser consumed the most memory, whereas Sealer took the longest time to finish. Considering the pros and cons of using TGS-GapCloser and Sealer involves considering a trade-off between resource efficiency and moderate accuracy.

RFfiller outperformed the gap fillers on the majority of datasets. RFfiller was primarily impacted by short gaps and an insufficient number of nucleotides on the flanks of the gap region. On short gaps (1 bp), the algorithm required a pattern within the candidate sequence. However, if the gap length is one base pair, all candidate sequences will be one base pair in length, and this makes it difficult to identify patterns within a sequence. On the other hand, when the gap length is greater than the length of either flank, the alignment generated on the gap region lacks sufficient information to extend the sequences as candidate sequences. On the SOAPdenovo2 scaffolds, this phenomenon was observed. These are the algorithm’s only limitations. Even in these instances, it generated fewer mismatches and misassemblies than the other gap fillers. According to our findings, the most effective method for filling in gaps is by using a statistical approach. Utilizing a hybrid method to update a draft *de novo* genome assembly, aided by RFfiller, is an efficient and accurate strategy for improving the quality of gene annotation and the structural variation detection. As a result, downstream analyses of high quality is possible.

##  Supplemental Information

10.7717/peerj.14186/supp-1 Supplemental Information 1 Supplemental Tables

## Additional Information and Declarations

### Data Availability

The following information was supplied regarding data availability: Data is available at RFfiller: Midekso, Firaol Dida, & Yi, Gangman. (2022). RFfiller. Zenodo. https://doi.org/10.5281/zenodo.7086759.
