# Supplementary material for: RFfiller: a robust and fast statistical algorithm for gap filling in draft genomes
Source: PeerJ. 2022 Oct 14;10:e14186. doi: 10.7717/peerj.14186 (PMC9575681; doi:10.7717/peerj.14186)
Supplement: Supplemental Information 1 [file peerj-10-14186-s001.pdf]

Supplementary Material: “Rffiller: A Robust And Fast Statistical Algorithm for Gap Filling in Draft Genome”.

Firaol Dida[1] and Gangman Yi[1,*]

[1]Department of Multimedia Engineering, Dongguk University, Seoul, 04620, Korea
*gangman@dongguk.edu

Table of Contents

Chapter 1 Genome assemblies **6**

A5-MiSeq *6*
Arabidopsis thaliana 6
Bacillus cereus 7
Escherichia coli 9
Homo sapien 11
Saccharomyces cerevisiae 13
Staphylococcus aureus 15

ABySS *17*
Arabidopsis thaliana 17
Bacillus cereus 18
Escherichia coli 20
Homo sapien 22
Saccharomyces cerevisiae 24
Staphylococcus aureus 25

FLYE *27*
Arabidopsis thaliana 27
Bacillus cereus 29
Escherichia coli 31
Homo sapien 33
Saccharomyces cerevisiae 34
Staphylococcus aureus 36

SGA *38*
Arabidopsis thaliana 38
Bacillus cereus 40
Escherichia coli 41
Homo sapien 43
Saccharomyces cerevisiae 45
Staphylococcus aureus 47

SOAPdenovo2 *49*
Arabidopsis thaliana 49
Bacillus cereus 50
Escherichia coli 52
Homo sapien 54
Saccharomyces cerevisiae 56
Staphylococcus aureus 58

SPAdes *60*
Arabidopsis thaliana 60
Bacillus cereus 61
Escherichia coli 63
Homo sapien 65
Saccharomyces cerevisiae 66
Staphylococcus aureus 68

Chapter 2 Scripts **70**

GapCloser *70*

Configuration file 70

Script 71

Sealer *71*

TGS-GapCloser *71*

Rffiller *71*

Chapter 3 Summary **71**

De novo assembly *71*

Gap fillers *73*

Table of Tables

Chapter 1 Genome assemblies **6**

A5-MiSeq *6*
Arabidopsis thaliana 6
Bacillus cereus 7
Escherichia coli 9
Homo sapien 11
Saccharomyces cerevisiae 13
Staphylococcus aureus 15

ABySS *17*
Arabidopsis thaliana 17
Bacillus cereus 18
Escherichia coli 20
Homo sapien 22
Saccharomyces cerevisiae 24
Staphylococcus aureus 25

FLYE *27*
Arabidopsis thaliana 27
Bacillus cereus 29
Escherichia coli 31
Homo sapien 33
Saccharomyces cerevisiae 34
Staphylococcus aureus 36

SGA *38*
Arabidopsis thaliana 38
Bacillus cereus 40
Escherichia coli 41
Homo sapien 43
Saccharomyces cerevisiae 45
Staphylococcus aureus 47

SOAPdenovo2 *49*
Arabidopsis thaliana 49
Bacillus cereus 50
Escherichia coli 52
Homo sapien 54
Saccharomyces cerevisiae 56
Staphylococcus aureus 58

SPAdes *60*
Arabidopsis thaliana 60
Bacillus cereus 61
Escherichia coli 63
Homo sapien 65
Saccharomyces cerevisiae 66
Staphylococcus aureus 68

Chapter 2 Summary **70**

De novo assembly *71*

Gap fillers *73*

Chapter 1 Genome assemblies

A5-MiSeq

Arabidopsis thaliana

Supplementary Table 1-1 Assembly statistics of gap filler on the Arabidopsis thaliana dataset. All statistics are based on contigs of size≥500 bp, unless otherwise noted (e.g., "# contigs(≥0 bp)" and "Total length (≥0 bp)" include all contigs).

Assembly	Scaffold	GapCloser	RFfiller	Sealer	TGS-GapCloser
# contigs (>= 0 bp)	33776	33776	33776	33776	33776
# contigs (>= 1000 bp)	570	570	570	569	570
# contigs (>= 5000 bp)	33	33	33	33	33
# contigs (>= 10000 bp)	13	13	13	13	13
# contigs (>= 25000 bp)	3	3	3	3	3
# contigs (>= 50000 bp)	1	1	1	1	1
Total length (>= 0 bp)	17245093	17245093	17245093	17238678	17245093
Total length (>= 1000 bp)	1146247	1146247	1146247	1145064	1146247
Total length (>= 5000 bp)	372626	372626	372626	372589	372626
Total length (>= 10000 bp)	236906	236906	236906	236879	236906
Total length (>= 25000 bp)	110774	110774	110774	110770	110774
Total length (>= 50000 bp)	55783	55783	55783	55781	55783
# contigs	11742	11742	11742	11716	11742
Largest contig	55783	55783	55783	55781	55783
Total length	8115734	8115734	8115734	8100945	8115734
Reference length	119668634	119668634	119668634	119668634	119668634
GC (%)	36.65	36.37	36.65	36.66	36.65
Reference GC (%)	36.06	36.06	36.06	36.06	36.06
N50	637	637	637	638	637
N75	555	555	555	555	555

**L50**	4453	4453	4453	4442	4453
**L75**	7878	7878	7878	7860	7878
**# misassemblies**	352	376	352	350	352
**# misassembled contigs**	345	367	345	343	345
**Misassembled contigs length**	316821	326346	316821	315744	316821
**# local misassemblies**	135	152	135	134	135
**# scaffold gap ext. mis.**	0	0	0	0	0
**# scaffold gap loc. mis.**	0	0	0	0	0
**# unaligned mis. contigs**	31	66	31	30	31
**# unaligned contigs**	192 + 31 part	340 + 117 part	192 + 31 part	192 + 31 part	192 + 31 part
**Unaligned length**	151128	331543	151128	151074	151128
**Genome fraction (%)**	6.491	6.312	6.491	6.484	6.491
**Duplication ratio**	1.027	1.032	1.027	1.026	1.027
**# N's per 100 kbp**	783.79	0	783.79	782.5	783.79
**# mismatches per 100 kbp**	429.5	762.17	429.5	425.66	429.5
**# indels per 100 kbp**	58.06	50.27	58.06	57.84	58.06
**Largest alignment**	55632	55632	55632	55630	55632
**Total aligned length**	7815659	7586395	7815659	7802685	7815659
**NA50**	618	609	618	618	618
**NGA50**	-	-	-	-	-
**NA75**	538	531	538	538	538
**LA50**	4636	4741	4636	4625	4636
**LA75**	8171	8332	8171	8152	8171

Bacillus cereus

Supplementary Table 1-2 Assembly statistics of gap filler on the Bacillus cereus dataset. All statistics are based on contigs of size≥500 bp, unless otherwise noted (e.g., "# contigs(≥0 bp)" and "Total length (≥0 bp)" include all contigs).

**Assembly**	**Scaffold**	**GapCloser**	**RFfiller**	**Sealer**	**TGS-GapCloser**

**# contigs (>= 0 bp)**	786	786	786	786	786
**# contigs (>= 1000 bp)**	689	689	689	689	689
**# contigs (>= 5000 bp)**	284	284	284	284	284
**# contigs (>= 10000 bp)**	144	144	144	144	144
**# contigs (>= 25000 bp)**	40	40	40	40	40
**# contigs (>= 50000 bp)**	11	11	11	11	11
**Total length (>= 0 bp)**	5275374	5275374	5275374	5275093	5275374
**Total length (>= 1000 bp)**	5198558	5198558	5198558	5198361	5198558
**Total length (>= 5000 bp)**	4193403	4193403	4193403	4193260	4193403
**Total length (>= 10000 bp)**	3208087	3208087	3208087	3207956	3208087
**Total length (>= 25000 bp)**	1650780	1650780	1650780	1650779	1650780
**Total length (>= 50000 bp)**	665759	665759	665759	665759	665759
**# contigs**	786	786	786	786	786
**Largest contig**	89286	89286	89286	89286	89286
**Total length**	5275374	5275374	5275374	5275093	5275374
**Reference length**	5427083	5427083	5427083	5427083	5427083
**GC (%)**	35.53	35.53	35.53	35.53	35.53
**Reference GC (%)**	35.29	35.29	35.29	35.29	35.29
**N50**	13922	13922	13922	13922	13922
**NG50**	13082	13082	13082	13082	13082
**N75**	6131	6131	6131	6131	6131
**NG75**	5546	5546	5546	5546	5546
**L50**	96	96	96	96	96
**LG50**	101	101	101	101	101
**L75**	241	241	241	241	241
**LG75**	261	261	261	261	261
**# misassemblies**	1	1	1	1	1
**# misassembled contigs**	1	1	1	1	1

**Misassembled contigs length**	1643	1643	1643	1643	1643
**# local misassemblies**	7	7	7	7	7
**# scaffold gap ext. mis.**	0	0	0	0	0
**# scaffold gap loc. mis.**	0	0	0	0	0
**# unaligned mis. contigs**	90	90	90	91	90
**# unaligned contigs**	552 + 218 part	551 + 219 part	552 + 218 part	551 + 218 part	552 + 218 part
**Unaligned length**	4950469	4951142	4950469	4949507	4950469
**Genome fraction (%)**	5.872	5.867	5.872	5.875	5.872
**Duplication ratio**	1.019	1.018	1.019	1.021	1.019
**# N's per 100 kbp**	15.15	0	15.15	14.99	15.15
**# mismatches per 100 kbp**	3746.24	3750.79	3746.24	3746.45	3746.24
**# indels per 100 kbp**	45.5	45.23	45.5	45.48	45.5
**Largest alignment**	31356	31356	31356	31356	31356
**Total aligned length**	321616	321724	321616	321757	321616
**NGA50**	-	-	-	-	-

Escherichia coli

Supplementary Table 1-3 Assembly statistics of gap filler on the Escherichia coli dataset. All statistics are based on contigs of size≥500 bp, unless otherwise noted (e.g., "# contigs(≥0 bp)" and "Total length (≥0 bp)" include all contigs).

**Assembly**	**Scaffold**	**GapCloser**	**RFfiller**	**Sealer**	**TGS-GapCloser**
**# contigs (>= 0 bp)**	280	280	280	280	280
**# contigs (>= 1000 bp)**	200	200	200	199	200
**# contigs (>= 5000 bp)**	102	102	102	102	102
**# contigs (>= 10000 bp)**	77	77	77	77	77
**# contigs (>= 25000 bp)**	54	54	54	54	54
**# contigs (>= 50000 bp)**	27	27	27	27	27
**Total length (>= 0 bp)**	5581170	5581170	5581170	5580605	5581170
**Total length (>= 1000 bp)**	5524100	5524100	5524100	5522867	5524100

**Total length (>= 5000 bp)**	5290187	5290187	5290187	5290144	5290187
**Total length (>= 10000 bp)**	5117036	5117036	5117036	5116997	5117036
**Total length (>= 25000 bp)**	4721582	4721582	4721582	4721554	4721582
**Total length (>= 50000 bp)**	3787497	3787497	3787497	3787487	3787497
**# contigs**	280	280	280	278	280
**Largest contig**	304243	304243	304243	304243	304243
**Total length**	5581170	5581170	5581170	5579718	5581170
**Reference length**	4641652	4641652	4641652	4641652	4641652
**GC (%)**	50.25	50.24	50.25	50.25	50.25
**Reference GC (%)**	50.79	50.79	50.79	50.79	50.79
**N50**	109023	109023	109023	109023	109023
**NG50**	136200	136200	136200	136200	136200
**N75**	36059	36059	36059	36058	36059
**NG75**	76735	76735	76735	76735	76735
**L50**	16	16	16	16	16
**LG50**	12	12	12	12	12
**L75**	37	37	37	37	37
**LG75**	23	23	23	23	23
**# misassemblies**	99	99	99	99	99
**# misassembled contigs**	39	39	39	39	39
**Misassembled contigs length**	3740666	3740666	3740666	3740654	3740666
**# local misassemblies**	154	154	154	154	154
**# scaffold gap ext. mis.**	0	0	0	0	0
**# scaffold gap loc. mis.**	0	0	0	0	0
**# unaligned mis. contigs**	19	19	19	19	19
**# unaligned contigs**	157 + 101 part	157 + 101 part	157 + 101 part	155 + 101 part	157 + 101 part
**Unaligned length**	1476242	1476641	1476242	1474814	1476242
**Genome fraction (%)**	87.809	87.809	87.809	87.808	87.809

**Duplication ratio**	1.007	1.007	1.007	1.007	1.007
**# N's per 100 kbp**	10	0	10	9.98	10
**# mismatches per 100 kbp**	1656.23	1659.34	1656.23	1656.21	1656.23
**# indels per 100 kbp**	23.48	23.48	23.48	23.48	23.48
**Largest alignment**	177388	177388	177388	177388	177388
**Total aligned length**	4096071	4096071	4096071	4096049	4096071
**NA50**	31211	31211	31211	31211	31211
**NGA50**	36307	36307	36307	36307	36307
**NGA75**	15999	15999	15999	15999	15999
**LA50**	48	48	48	48	48
**LGA50**	33	33	33	33	33
**LGA75**	77	77	77	77	77

Homo sapien

Supplementary Table 1-4 Assembly statistics of gap filler on the Homo sapien dataset. All statistics are based on contigs of size≥500 bp, unless otherwise noted (e.g., ”# contigs(≥0 bp)” and ”Total length (≥0 bp)” include all contigs).

**Assembly**	**Scaffold**	**GapCloser**	**RFfiller**	**Sealer**	**TGS-GapCloser**
**# contigs (>= 0 bp)**	748312	748312	748312	748312	748312
**# contigs (>= 1000 bp)**	5045	5045	5045	5042	5045
**# contigs (>= 5000 bp)**	152	152	152	152	152
**# contigs (>= 10000 bp)**	15	15	15	15	15
**# contigs (>= 25000 bp)**	2	2	2	2	2
**# contigs (>= 50000 bp)**	2	2	2	2	2
**Total length (>= 0 bp)**	314253487	314253499	314253487	314167420	314253396
**Total length (>= 1000 bp)**	9037257	9037263	9037257	9031701	9037206
**Total length (>= 5000 bp)**	1196444	1196445	1196444	1196307	1196412
**Total length (>= 10000 bp)**	315767	315767	315767	315736	315778
**Total length (>= 25000**	131762	131762	131762	131760	131773

**bp)**					
**Total length (>= 50000 bp)**	131762	131762	131762	131760	131773
**# contigs**	120404	120404	120404	120274	120404
**Largest contig**	73731	73731	73731	73731	73731
**Total length**	78527682	78527694	78527682	78444601	78527591
**Reference length**	3272089205	3272089205	3272089205	3272089205	3272089205
**GC (%)**	40.62	40.26	40.62	40.62	40.62
**Reference GC (%)**	41.04	41.04	41.04	41.04	41.04
**N50**	602	602	602	602	602
**N75**	540	540	540	540	540
**L50**	47982	47982	47982	47928	47982
**L75**	82538	82538	82538	82448	82538
**# misassemblies**	5393	5955	5393	5381	5393
**# misassembled contigs**	5237	5713	5237	5225	5237
**Misassembled contigs length**	3691016	4125338	3691016	3681848	3691016
**# local misassemblies**	275	394	275	273	275
**# scaffold gap ext. mis.**	0	0	0	0	0
**# scaffold gap loc. mis.**	0	0	0	0	0
**# unaligned mis. contigs**	101	336	101	103	101
**# unaligned contigs**	1446 + 217 part	1984 + 681 part	1446 + 217 part	1439 + 215 part	1446 + 217 part
**Unaligned length**	1500015	2354462	1500020	1492481	1500031
**Genome fraction (%)**	2.439	2.401	2.439	2.436	2.439
**Duplication ratio**	1.015	1.02	1.015	1.015	1.015
**# N's per 100 kbp**	888.75	0.11	888.6	888.8	888.52
**# mismatches per 100 kbp**	233.54	702.99	233.54	232.97	233.55
**# indels per 100 kbp**	34.47	28.37	34.48	34.45	34.47
**Largest alignment**	18785	18785	18785	18785	18785
**Total aligned length**	75974294	74787610	75974256	75904734	75974192

**NA50**	584	579	584	584	584
**NGA50**	-	-	-	-	-
**NA75**	526	522	526	526	526
**LA50**	50400	51355	50400	50337	50400
**LA75**	85982	87209	85982	85876	85982

Saccharomyces cerevisiae

Supplementary Table 1-5 Assembly statistics of gap filler on the Saccharomyces cerevisiae dataset. All statistics are based on contigs of size≥500 bp, unless otherwise noted (e.g., "# contigs(≥0 bp)" and "Total length (≥0 bp)" include all contigs).

**Assembly**	**Scaffold**	**GapCloser**	**RFfiller**	**Sealer**	**TGS-GapCloser**
**# contigs (>= 0 bp)**	670	670	670	670	670
**# contigs (>= 1000 bp)**	263	263	263	263	263
**# contigs (>= 5000 bp)**	194	194	194	194	194
**# contigs (>= 10000 bp)**	164	164	164	164	164
**# contigs (>= 25000 bp)**	127	127	127	127	127
**# contigs (>= 50000 bp)**	86	86	86	86	86
**Total length (>= 0 bp)**	11671453	11671747	11671453	11671267	11679008
**Total length (>= 1000 bp)**	11504571	11504865	11504571	11504532	11512126
**Total length (>= 5000 bp)**	11349731	11350024	11349731	11349711	11357248
**Total length (>= 10000 bp)**	11139995	11140286	11139995	11139977	11147372
**Total length (>= 25000 bp)**	10489093	10489384	10489093	10489103	10496470
**Total length (>= 50000 bp)**	9029598	9029885	9029598	9029588	9031078
**# contigs**	369	369	369	369	369
**Largest contig**	292060	292060	292060	292060	292072
**Total length**	11574768	11575062	11574768	11574724	11582323
**Reference length**	12157105	12157105	12157105	12157105	12157105
**GC (%)**	38.12	38.1	38.12	38.12	38.12
**Reference GC (%)**	38.15	38.15	38.15	38.15	38.15
**N50**	91325	91325	91325	91325	91325

**NG50**	87302	87302	87302	87282	87302
**N75**	55570	55570	55570	55570	55570
**NG75**	46980	46980	46980	46980	46980
**L50**	39	39	39	39	39
**LG50**	42	42	42	42	42
**L75**	80	80	80	80	80
**LG75**	88	88	88	88	88
**# misassemblies**	71	68	71	70	70
**# misassembled contigs**	54	51	54	53	53
**Misassembled contigs length**	3412296	3408196	3412296	3403239	3437035
**# local misassemblies**	98	109	105	97	101
**# scaffold gap ext. mis.**	0	0	0	0	0
**# scaffold gap loc. mis.**	7	0	0	7	4
**# unaligned mis. contigs**	14	14	14	14	14
**# unaligned contigs**	39 + 29 part	39 + 29 part	39 + 29 part	39 + 29 part	39 + 32 part
**Unaligned length**	123111	123455	123146	122373	130332
**Genome fraction (%)**	93.838	93.839	93.838	93.839	93.839
**Duplication ratio**	1.004	1.004	1.004	1.004	1.004
**# N's per 100 kbp**	53.02	0.14	49.13	51.88	49.81
**# mismatches per 100 kbp**	559.16	593.2	559.23	559.85	559.51
**# indels per 100 kbp**	50.5	50.53	50.59	50.6	50.92
**Largest alignment**	238989	238989	238989	238989	238989
**Total aligned length**	11423212	11423302	11423212	11423779	11424071
**NA50**	77574	77574	77574	77574	77574
**NGA50**	72006	72006	72006	72006	72006
**NA75**	40615	40615	40615	40615	40615
**NGA75**	36340	36340	36340	36340	36340
**LA50**	48	48	48	48	48
**LGA50**	52	52	52	52	52
**LA75**	98	98	98	98	98

LGA75	109	109	109	109	109

Staphylococcus aureus

Supplementary Table 1-6 Assembly statistics of gap filler on the Staphylococcus aureus dataset. All statistics are based on contigs of size≥500 bp, unless otherwise noted (e.g., "# contigs(≥0 bp)" and "Total length (≥0 bp)" include all contigs).

Assembly	Scaffold	GapCloser	RFfiller	Sealer	TGS-GapCloser
# contigs (>= 0 bp)	36	36	36	36	36
# contigs (>= 1000 bp)	28	28	28	28	28
# contigs (>= 5000 bp)	21	21	21	21	21
# contigs (>= 10000 bp)	21	21	21	21	21
# contigs (>= 25000 bp)	20	20	20	20	20
# contigs (>= 50000 bp)	15	15	15	15	15
Total length (>= 0 bp)	2799146	2799146	2799146	2798834	2799146
Total length (>= 1000 bp)	2793070	2793070	2793070	2792778	2793070
Total length (>= 5000 bp)	2776324	2776324	2776324	2776276	2776324
Total length (>= 10000 bp)	2776324	2776324	2776324	2776276	2776324
Total length (>= 25000 bp)	2759350	2759350	2759350	2759302	2759350
Total length (>= 50000 bp)	2571882	2571882	2571882	2571836	2571882
# contigs	36	36	36	36	36
Largest contig	450615	450615	450615	450615	450615
Total length	2799146	2799146	2799146	2798834	2799146
Reference length	2821361	2821361	2821361	2821361	2821361
GC (%)	32.69	32.69	32.69	32.69	32.69
Reference GC (%)	32.87	32.87	32.87	32.87	32.87
N50	244991	244991	244991	244990	244991
NG50	177125	177125	177125	177123	177125
N75	108646	108646	108646	108646	108646
NG75	107881	107881	107881	107880	107881
L50	4	4	4	4	4

**LG50**	5	5	5	5	5
**L75**	9	9	9	9	9
**LG75**	10	10	10	10	10
**# misassemblies**	67	67	67	67	67
**# misassembled contigs**	19	19	19	19	19
**Misassembled contigs length**	2657100	2657100	2657100	2657053	2657100
**# local misassemblies**	93	93	93	92	93
**# scaffold gap ext. mis.**	0	0	0	0	0
**# scaffold gap loc. mis.**	0	0	0	0	0
**# unaligned mis. contigs**	2	2	2	2	2
**# unaligned contigs**	2 + 18 part	2 + 18 part	2 + 18 part	2 + 18 part	2 + 18 part
**Unaligned length**	276955	276980	276955	276910	276955
**Genome fraction (%)**	88.847	88.847	88.847	88.861	88.847
**Duplication ratio**	1.006	1.006	1.006	1.006	1.006
**# N's per 100 kbp**	1.43	0	1.43	1.43	1.43
**# mismatches per 100 kbp**	1393.74	1394.22	1393.74	1393.45	1393.74
**# indels per 100 kbp**	49.15	49.15	49.15	49.14	49.15
**Largest alignment**	171163	171163	171163	171163	171163
**Total aligned length**	2518210	2518210	2518210	2517945	2518210
**NA50**	72014	72014	72014	72014	72014
**NGA50**	72014	72014	72014	72014	72014
**NA75**	23621	23621	23621	23621	23621
**NGA75**	23441	23441	23441	23441	23441
**LA50**	13	13	13	13	13
**LGA50**	13	13	13	13	13
**LA75**	30	30	30	30	30
**LGA75**	31	31	31	31	31

ABySS

Arabidopsis thaliana

Supplementary Table 1-7 Assembly statistics of gap filler on the Arabidopsis thaliana dataset. All statistics are based on contigs of size≥500 bp, unless otherwise noted (e.g., "# contigs(≥0 bp)" and "Total length (≥0 bp)" include all contigs).

Assembly	Scaffold	GapCloser	RFfiller	Sealer	TGS-GapCloser
# contigs (>= 0 bp)	171343	171343	171343	171343	171343
# contigs (>= 1000 bp)	120	120	120	120	120
# contigs (>= 5000 bp)	0	0	0	0	0
# contigs (>= 10000 bp)	0	0	0	0	0
# contigs (>= 25000 bp)	0	0	0	0	0
# contigs (>= 50000 bp)	0	0	0	0	0
Total length (>= 0 bp)	28039698	28039698	28039698	28039698	28039698
Total length (>= 1000 bp)	213290	213290	213290	213290	213290
Total length (>= 5000 bp)	0	0	0	0	0
Total length (>= 10000 bp)	0	0	0	0	0
Total length (>= 25000 bp)	0	0	0	0	0
Total length (>= 50000 bp)	0	0	0	0	0
# contigs	855	855	855	855	855
Largest contig	4444	4444	4444	4444	4444
Total length	662354	662354	662354	662354	662354
Reference length	119668634	119668634	119668634	119668634	119668634
GC (%)	39.97	39.97	39.97	39.97	39.97
Reference GC (%)	36.06	36.06	36.06	36.06	36.06
N50	687	687	687	687	687
N75	554	554	554	554	554
L50	268	268	268	268	268
L75	540	540	540	540	540
# misassemblies	12	12	12	12	12
# misassembled contigs	12	12	12	12	12

**Misassembled contigs length**	9243	9243	9243	9243	9243
**# local misassemblies**	3	3	3	3	3
**# scaffold gap ext. mis.**	0	0	0	0	0
**# scaffold gap loc. mis.**	0	0	0	0	0
**# unaligned mis. contigs**	2	2	2	2	2
**# unaligned contigs**	17 + 0 part	17 + 0 part	17 + 0 part	17 + 0 part	17 + 0 part
**Unaligned length**	9582	9582	9582	9582	9582
**Genome fraction (%)**	0.541	0.541	0.541	0.541	0.541
**Duplication ratio**	1.009	1.009	1.009	1.009	1.009
**# N's per 100 kbp**	0	0	0	0	0
**# mismatches per 100 kbp**	196.98	196.98	196.98	196.98	196.98
**# indels per 100 kbp**	21.18	21.18	21.18	21.18	21.18
**Largest alignment**	4444	4444	4444	4444	4444
**Total aligned length**	648770	648770	648770	648770	648770
**NA50**	683	683	683	683	683
**NGA50**	-	-	-	-	-
**NA75**	548	548	548	548	548
**LA50**	270	270	270	270	270
**LA75**	545	545	545	545	545

Bacillus cereus

Supplementary Table 1-8 Assembly statistics of gap filler on the Bacillus cereus dataset. All statistics are based on contigs of size≥500 bp, unless otherwise noted (e.g., "# contigs(≥0 bp)" and "Total length (≥0 bp)" include all contigs).

**Assembly**	**Scaffold**	**GapCloser**	**RFfiller**	**Sealer**	**TGS-GapCloser**
**# contigs (>= 0 bp)**	1529	1529	1529	1529	1529
**# contigs (>= 1000 bp)**	617	617	617	617	617
**# contigs (>= 5000 bp)**	260	260	260	260	260
**# contigs (>= 10000 bp)**	142	142	142	143	143
**# contigs (>= 25000 bp)**	42	42	42	42	42

**# contigs (>= 50000 bp)**	11	11	11	11	11
**Total length (>= 0 bp)**	5309127	5309079	5309127	5308940	5311845
**Total length (>= 1000 bp)**	5092001	5091953	5092001	5091814	5094719
**Total length (>= 5000 bp)**	4212736	4212686	4212736	4212874	4215454
**Total length (>= 10000 bp)**	3374857	3374806	3374857	3384858	3387438
**Total length (>= 25000 bp)**	1779823	1779826	1779823	1779921	1779620
**Total length (>= 50000 bp)**	756792	756793	756792	756718	756792
**# contigs**	742	742	742	742	742
**Largest contig**	92775	92775	92775	92693	92775
**Total length**	5185929	5185881	5185929	5185742	5188647
**Reference length**	5427083	5427083	5427083	5427083	5427083
**GC (%)**	35.52	35.53	35.53	35.53	35.52
**Reference GC (%)**	35.29	35.29	35.29	35.29	35.29
**N50**	16823	16823	16823	16823	16823
**NG50**	15968	15968	15968	15968	15968
**N75**	6856	6856	6856	6856	6856
**NG75**	5627	5627	5627	5627	5627
**L50**	82	82	82	82	82
**LG50**	90	90	90	90	90
**L75**	204	204	204	204	204
**LG75**	233	233	233	233	233
**# misassemblies**	1	1	1	1	1
**# misassembled contigs**	1	1	1	1	1
**Misassembled contigs length**	3390	3390	3390	3390	3390
**# local misassemblies**	3	3	3	3	3
**# scaffold gap ext. mis.**	0	0	0	0	0
**# scaffold gap loc. mis.**	0	0	0	0	0
**# unaligned mis. contigs**	93	93	93	93	93

# unaligned contigs	522 + 216 part	522 + 216 part	522 + 216 part	522 + 216 part	522 + 216 part
Unaligned length	4877217	4877610	4877501	4877625	4880035
Genome fraction (%)	5.57	5.578	5.571	5.574	5.57
Duplication ratio	1.021	1.018	1.02	1.019	1.021
# N's per 100 kbp	28.29	11.8	22.58	13.07	26.35
# mismatches per 100 kbp	3870.2	3870.27	3872.36	3869.7	3870.2
# indels per 100 kbp	24.15	24.45	24.15	24.13	24.15
Largest alignment	30846	30846	30846	30846	30846
Total aligned length	306444	306853	306456	306638	306444
NGA50	-	-	-	-	-

Escherichia coli

Supplementary Table 1-9 Assembly statistics of gap filler on the Escherichia coli dataset. All statistics are based on contigs of size≥500 bp, unless otherwise noted (e.g., "# contigs(≥0 bp)" and "Total length (≥0 bp)" include all contigs).

Assembly	Scaffold	GapCloser	RFfiller	Sealer	TGS-GapCloser
# contigs (>= 0 bp)	1342	1342	1342	1342	1342
# contigs (>= 1000 bp)	242	242	242	242	242
# contigs (>= 5000 bp)	154	154	154	155	154
# contigs (>= 10000 bp)	115	115	115	115	115
# contigs (>= 25000 bp)	67	67	67	67	67
# contigs (>= 50000 bp)	24	24	24	24	24
Total length (>= 0 bp)	5553067	5553262	5553067	5553815	5558672
Total length (>= 1000 bp)	5334134	5334329	5334134	5334882	5339739
Total length (>= 5000 bp)	5100988	5101178	5100988	5106110	5106593
Total length (>= 10000 bp)	4806232	4806417	4806232	4806251	4811837
Total length (>= 25000 bp)	3986629	3986633	3986629	3986695	3992225
Total length (>= 50000	2390036	2390037	2390036	2390037	2391398

**bp)**					
**# contigs**	324	324	324	324	324
**Largest contig**	182191	182191	182191	182191	182191
**Total length**	5391592	5391787	5391592	5392340	5397197
**Reference length**	4641652	4641652	4641652	4641652	4641652
**GC (%)**	50.29	50.29	50.29	50.3	50.29
**Reference GC (%)**	50.79	50.79	50.79	50.79	50.79
**N50**	46745	46745	46745	46745	46932
**NG50**	51892	51892	51892	51892	51892
**N75**	24822	24822	24822	24822	24822
**NG75**	33053	33053	33053	33053	33053
**L50**	31	31	31	31	31
**LG50**	23	23	23	23	23
**L75**	70	70	70	70	70
**LG75**	50	50	50	50	50
**# misassemblies**	82	84	83	83	82
**# misassembled contigs**	57	57	57	57	57
**Misassembled contigs length**	3058020	3058023	3058020	3058087	3063619
**# local misassemblies**	130	132	132	132	133
**# scaffold gap ext. mis.**	2	0	1	1	0
**# scaffold gap loc. mis.**	2	1	1	0	1
**# unaligned mis. contigs**	11	11	11	11	11
**# unaligned contigs**	164 + 105 part	164 + 104 part	164 + 104 part	164 + 104 part	164 + 105 part
**Unaligned length**	1280585	1280950	1280054	1281097	1284538
**Genome fraction (%)**	87.679	87.69	87.685	87.691	87.72
**Duplication ratio**	1.01	1.01	1.01	1.01	1.01
**# N's per 100 kbp**	20.72	3.06	15.38	7.4	16.95
**# mismatches per 100 kbp**	1645.47	1645.53	1645.71	1645.72	1645.9
**# indels per 100 kbp**	23.17	23.22	23.37	23.17	26.28
**Largest alignment**	177283	177283	177283	177283	177283

**Total aligned length**	4098089	4098461	4098267	4098645	4100013
**NA50**	26242	26242	26242	26242	26242
**NGA50**	32234	31906	31906	31906	32234
**NA75**	1466	1466	1466	1466	1436
**NGA75**	12781	12781	12781	12781	12850
**LA50**	56	56	56	56	56
**LGA50**	43	43	43	43	43
**LA75**	186	188	187	187	188
**LGA75**	99	99	99	99	98

Homo sapien

Supplementary Table 1-10 Assembly statistics of gap filler on the Homo sapien dataset. All statistics are based on contigs of size≥500 bp, unless otherwise noted (e.g., "# contigs(≥0 bp)" and "Total length (≥0 bp)" include all contigs).

**Assembly**	**Scaffold**	**GapCloser**	**RFfiller**	**Sealer**	**TGS-GapCloser**
**# contigs (>= 0 bp)**	449109	449109	449109	449109	449109
**# contigs (>= 1000 bp)**	12	12	12	12	12
**# contigs (>= 5000 bp)**	0	0	0	0	0
**# contigs (>= 10000 bp)**	0	0	0	0	0
**# contigs (>= 25000 bp)**	0	0	0	0	0
**# contigs (>= 50000 bp)**	0	0	0	0	0
**Total length (>= 0 bp)**	13354571	13354571	13354571	13354571	13354571
**Total length (>= 1000 bp)**	22356	22356	22356	22356	22356
**Total length (>= 5000 bp)**	0	0	0	0	0
**Total length (>= 10000 bp)**	0	0	0	0	0
**Total length (>= 25000 bp)**	0	0	0	0	0
**Total length (>= 50000 bp)**	0	0	0	0	0
**# contigs**	46	46	46	46	46
**Largest contig**	4764	4764	4764	4764	4764
**Total length**	46209	46209	46209	46209	46209
**Reference length**	327208920	327208920	3272089205	327208920	3272089205

	5	5		5	
**GC (%)**	54.03	54.03	54.03	54.03	54.03
**Reference GC (%)**	41.04	41.04	41.04	41.04	41.04
**N50**	980	980	980	980	980
**N75**	704	704	704	704	704
**L50**	13	13	13	13	13
**L75**	27	27	27	27	27
**# misassemblies**	0	0	0	0	0
**# misassembled contigs**	0	0	0	0	0
**Misassembled contigs length**	0	0	0	0	0
**# local misassemblies**	0	0	0	0	0
**# scaffold gap ext. mis.**	0	0	0	0	0
**# scaffold gap loc. mis.**	0	0	0	0	0
**# unaligned mis. contigs**	0	0	0	0	0
**# unaligned contigs**	3 + 0 part	3 + 0 part	3 + 0 part	3 + 0 part	3 + 0 part
**Unaligned length**	3350	3350	3350	3350	3350
**Genome fraction (%)**	0.001	0.001	0.001	0.001	0.001
**Duplication ratio**	1.005	1.005	1.005	1.005	1.005
**# N's per 100 kbp**	0	0	0	0	0
**# mismatches per 100 kbp**	227.45	227.45	227.45	227.45	227.45
**# indels per 100 kbp**	18.76	18.76	18.76	18.76	18.76
**Largest alignment**	4764	4764	4764	4764	4764
**Total aligned length**	42834	42834	42834	42834	42834
**NA50**	916	916	916	916	916
**NGA50**	-	-	-	-	-
**NA75**	662	662	662	662	662
**LA50**	15	15	15	15	15
**LA75**	29	29	29	29	29

Saccharomyces cerevisiae

Supplementary Table 1-11 Assembly statistics of gap filler on the Saccharomyces cerevisiae dataset. All statistics are based on contigs of size≥500 bp, unless otherwise noted (e.g., "# contigs(≥0 bp)" and "Total length (≥0 bp)" include all contigs).

Assembly	Scaffold	GapCloser	RFfiller	Sealer	TGS-GapCloser
# contigs (>= 0 bp)	1823	1823	1823	1823	1823
# contigs (>= 1000 bp)	226	226	226	226	226
# contigs (>= 5000 bp)	154	154	154	154	154
# contigs (>= 10000 bp)	140	140	140	140	140
# contigs (>= 25000 bp)	115	115	115	115	115
# contigs (>= 50000 bp)	81	81	81	81	81
Total length (>= 0 bp)	11746805	11748259	11746805	11749832	11769062
Total length (>= 1000 bp)	11481709	11483163	11481709	11484736	11503966
Total length (>= 5000 bp)	11276191	11277644	11276191	11279218	11298448
Total length (>= 10000 bp)	11167833	11169286	11167833	11170860	11190090
Total length (>= 25000 bp)	10727514	10728965	10727514	10730482	10749711
Total length (>= 50000 bp)	9432484	9433928	9432484	9434697	9453937
# contigs	271	271	271	271	271
Largest contig	381944	381945	381944	381936	381938
Total length	11514076	11515530	11514076	11517103	11536333
Reference length	12157105	12157105	12157105	12157105	12157105
GC (%)	38.11	38.11	38.11	38.11	38.12
Reference GC (%)	38.15	38.15	38.15	38.15	38.15
N50	109442	109567	109442	109566	109558
NG50	103986	103987	103986	104007	104002
N75	60512	60512	60512	60512	60512
NG75	54763	56841	54763	56841	56841
L50	32	32	32	32	32
LG50	35	35	35	35	35
L75	67	67	67	67	67
LG75	76	75	76	75	75

**# misassemblies**	64	67	66	64	66
**# misassembled contigs**	47	47	47	46	46
**Misassembled contigs length**	4591897	4593558	4591897	4467479	4487585
**# local misassemblies**	83	98	90	84	86
**# scaffold gap ext. mis.**	3	0	1	2	0
**# scaffold gap loc. mis.**	28	3	21	6	3
**# unaligned mis. contigs**	6	6	6	6	6
**# unaligned contigs**	7 + 24 part	7 + 24 part	7 + 24 part	7 + 24 part	7 + 27 part
**Unaligned length**	82782	82982	82923	82782	102207
**Genome fraction (%)**	93.444	93.477	93.445	93.485	93.483
**Duplication ratio**	1.006	1.006	1.006	1.006	1.006
**# N's per 100 kbp**	44.09	2.81	30.25	14.83	8.15
**# mismatches per 100 kbp**	580.03	580.6	580.89	580.47	582.72
**# indels per 100 kbp**	48.9	48.98	49.23	48.82	53.57
**Largest alignment**	274737	274737	274737	274737	274737
**Total aligned length**	11408087	11410118	11408091	11411158	11412329
**NA50**	94568	94648	94568	94647	93565
**NGA50**	92224	92225	92224	92305	89077
**NA75**	49204	46496	46155	49204	45121
**NGA75**	40950	40429	40429	41124	40316
**LA50**	41	41	41	41	41
**LGA50**	44	44	44	44	45
**LA75**	83	83	83	83	85
**LGA75**	94	95	95	93	96

Staphylococcus aureus

Supplementary Table 1-12 Assembly statistics of gap filler on the Staphylococcus aureus dataset. All statistics are based on contigs of size≥500 bp, unless otherwise noted (e.g., "# contigs(≥0 bp)" and "Total length (≥0 bp)" include all contigs).

Assembly	Scaffold	GapCloser	RFfiller	Sealer	TGS-GapCloser
**# contigs (>= 0 bp)**	269	269	269	269	269
**# contigs (>= 1000 bp)**	53	53	53	53	53
**# contigs (>= 5000 bp)**	46	46	46	46	46
**# contigs (>= 10000 bp)**	41	41	41	41	41
**# contigs (>= 25000 bp)**	31	31	31	31	31
**# contigs (>= 50000 bp)**	19	19	19	19	19
**Total length (>= 0 bp)**	2831214	2831222	2831214	2831100	2835012
**Total length (>= 1000 bp)**	2797816	2797824	2797816	2797702	2801614
**Total length (>= 5000 bp)**	2774906	2774914	2774906	2774792	2778704
**Total length (>= 10000 bp)**	2731082	2731090	2731082	2730968	2734880
**Total length (>= 25000 bp)**	2570893	2570901	2570893	2570779	2574691
**Total length (>= 50000 bp)**	2129221	2129227	2129221	2129124	2133019
**# contigs**	60	60	60	60	60
**Largest contig**	260661	260661	260661	260661	260661
**Total length**	2802984	2802992	2802984	2802870	2806782
**Reference length**	2821361	2821361	2821361	2821361	2821361
**GC (%)**	32.62	32.62	32.62	32.62	32.62
**Reference GC (%)**	32.87	32.87	32.87	32.87	32.87
**N50**	92586	92587	92586	92488	92488
**NG50**	92586	92587	92586	92488	92488
**N75**	55762	55763	55762	55762	55762
**NG75**	55762	55763	55762	55762	55762
**L50**	10	10	10	10	10
**LG50**	10	10	10	10	10
**L75**	19	19	19	19	19
**LG75**	19	19	19	19	19
**# misassemblies**	42	42	42	42	44
**# misassembled contigs**	25	25	25	25	25

**Misassembled contigs length**	2086123	2086129	2086123	2086016	2089990
**# local misassemblies**	79	83	80	82	80
**# scaffold gap ext. mis.**	0	0	0	0	0
**# scaffold gap loc. mis.**	3	0	2	0	0
**# unaligned mis. contigs**	3	3	3	3	3
**# unaligned contigs**	2 + 39 part	2 + 39 part	2 + 39 part	2 + 39 part	2 + 39 part
**Unaligned length**	304224	304307	304279	304075	307781
**Genome fraction (%)**	88.266	88.27	88.266	88.274	88.278
**Duplication ratio**	1.003	1.003	1.003	1.003	1.003
**# N's per 100 kbp**	7.67	0.32	5.71	0.07	1.96
**# mismatches per 100 kbp**	1390.48	1390.53	1390.48	1390.5	1391.69
**# indels per 100 kbp**	48.15	48.14	48.15	48.22	48.86
**Largest alignment**	171163	171163	171163	171163	171163
**Total aligned length**	2495696	2495826	2495696	2495943	2496097
**NA50**	55411	55411	55411	55411	55411
**NGA50**	55411	55411	55411	55411	55411
**NA75**	21757	21757	21757	21757	21757
**NGA75**	21757	21757	21757	21757	19037
**LA50**	15	15	15	15	15
**LGA50**	15	15	15	15	15
**LA75**	36	36	36	36	36
**LGA75**	36	36	36	36	37

FLYE

Arabidopsis thaliana

Supplementary Table 1-13 Assembly statistics of gap filler on the Arabidopsis thaliana dataset. All statistics are based on contigs of size≥500 bp, unless otherwise noted (e.g., "# contigs(≥0 bp)" and "Total length (≥0 bp)" include all contigs).

Assembly	Scaffold	GapCloser	RFfiller	Sealer	TGS-GapCloser
**# contigs (>= 0 bp)**	1832	1832	1832	1832	1832
**# contigs (>= 1000 bp)**	1797	1797	1797	1797	1797
**# contigs (>= 5000 bp)**	1753	1753	1753	1753	1753
**# contigs (>= 10000 bp)**	1667	1667	1667	1667	1667
**# contigs (>= 25000 bp)**	1424	1424	1424	1424	1424
**# contigs (>= 50000 bp)**	846	846	846	846	846
**Total length (>= 0 bp)**	105581485	105581485	105581485	105581485	105581485
**Total length (>= 1000 bp)**	105557593	105557593	105557593	105557593	105557593
**Total length (>= 5000 bp)**	105421149	105421149	105421149	105421149	105421149
**Total length (>= 10000 bp)**	104748508	104748508	104748508	104748508	104748508
**Total length (>= 25000 bp)**	100581083	100581083	100581083	100581083	100581083
**Total length (>= 50000 bp)**	78560947	78560947	78560947	78560947	78560947
**# contigs**	1832	1832	1832	1832	1832
**Largest contig**	404821	404821	404821	404821	404821
**Total length**	105581485	105581485	105581485	105581485	105581485
**Reference length**	119668634	119668634	119668634	119668634	119668634
**GC (%)**	37.24	37.24	37.24	37.24	37.24
**Reference GC (%)**	36.06	36.06	36.06	36.06	36.06
**N50**	77191	77191	77191	77191	77191
**NG50**	68698	68698	68698	68698	68698
**N75**	49468	49468	49468	49468	49468
**NG75**	39576	39576	39576	39576	39576
**L50**	432	432	432	432	432
**LG50**	529	529	529	529	529
**L75**	859	859	859	859	859
**LG75**	1096	1096	1096	1096	1096

**# misassemblies**	2562	2562	2562	2562	2562
**# misassembled contigs**	869	869	869	869	869
**Misassembled contigs length**	65888898	65888898	65888898	65888898	65888898
**# local misassemblies**	17865	17865	17865	17865	17865
**# scaffold gap ext. mis.**	0	0	0	0	0
**# scaffold gap loc. mis.**	1	1	1	1	1
**# unaligned mis. contigs**	240	240	240	240	240
**# unaligned contigs**	0 + 1492 part	0 + 1492 part	0 + 1492 part	0 + 1492 part	0 + 1492 part
**Unaligned length**	17688341	17688341	17688341	17688341	17688341
**Genome fraction (%)**	71.521	71.521	71.521	71.521	71.521
**Duplication ratio**	1.029	1.029	1.029	1.029	1.029
**# N's per 100 kbp**	0.19	0.19	0.19	0.19	0.19
**# mismatches per 100 kbp**	1581.86	1581.86	1581.86	1581.86	1581.86
**# indels per 100 kbp**	1682.32	1682.32	1682.32	1682.32	1682.32
**Largest alignment**	323229	323229	323229	323229	323229
**Total aligned length**	87766020	87766020	87766020	87766020	87766020
**NA50**	37896	37896	37896	37896	37896
**NGA50**	31486	31486	31486	31486	31486
**NA75**	9919	9919	9919	9919	9919
**LA50**	788	788	788	788	788
**LGA50**	991	991	991	991	991
**LA75**	2008	2008	2008	2008	2008

Bacillus cereus

Supplementary Table 1-14 Assembly statistics of gap filler on the Bacillus cereus dataset. All statistics are based on contigs of size≥500 bp, unless otherwise noted (e.g., "# contigs(≥0 bp)" and "Total length (≥0 bp)" include all contigs).

**Assembly**	**Scaffold**	**GapCloser**	**RFfiller**	**Sealer**	**TGS-GapCloser**
**# contigs (>= 0 bp)**	15	15	15	15	15

**# contigs (>= 1000 bp)**	15	15	15	15	15
**# contigs (>= 5000 bp)**	15	15	15	15	15
**# contigs (>= 10000 bp)**	14	14	14	14	14
**# contigs (>= 25000 bp)**	5	5	5	5	5
**# contigs (>= 50000 bp)**	5	5	5	5	5
**Total length (>= 0 bp)**	5747445	5747445	5747445	5747445	5747445
**Total length (>= 1000 bp)**	5747445	5747445	5747445	5747445	5747445
**Total length (>= 5000 bp)**	5747445	5747445	5747445	5747445	5747445
**Total length (>= 10000 bp)**	5738480	5738480	5738480	5738480	5738480
**Total length (>= 25000 bp)**	5610921	5610921	5610921	5610921	5610921
**Total length (>= 50000 bp)**	5610921	5610921	5610921	5610921	5610921
**# contigs**	15	15	15	15	15
**Largest contig**	3779869	3779869	3779869	3779869	3779869
**Total length**	5747445	5747445	5747445	5747445	5747445
**Reference length**	5427083	5427083	5427083	5427083	5427083
**GC (%)**	35.29	35.29	35.29	35.29	35.29
**Reference GC (%)**	35.29	35.29	35.29	35.29	35.29
**N50**	3779869	3779869	3779869	3779869	3779869
**NG50**	3779869	3779869	3779869	3779869	3779869
**N75**	1269365	1269365	1269365	1269365	1269365
**NG75**	1269365	1269365	1269365	1269365	1269365
**L50**	1	1	1	1	1
**LG50**	1	1	1	1	1
**L75**	2	2	2	2	2
**LG75**	2	2	2	2	2
**# misassemblies**	5	5	5	5	5
**# misassembled contigs**	1	1	1	1	1
**Misassembled contigs length**	197167	197167	197167	197167	197167
**# local misassemblies**	16	16	16	16	16
**# scaffold gap ext. mis.**	0	0	0	0	0
**# scaffold gap loc. mis.**	0	0	0	0	0

**# unaligned mis. contigs**	5	5	5	5	5
**# unaligned contigs**	5 + 10 part	5 + 10 part	5 + 10 part	5 + 10 part	5 + 10 part
**Unaligned length**	4759365	4759365	4759365	4759365	4759365
**Genome fraction (%)**	17.829	17.829	17.829	17.829	17.829
**Duplication ratio**	1.021	1.021	1.021	1.021	1.021
**# N's per 100 kbp**	0	0	0	0	0
**# mismatches per 100 kbp**	3604.16	3604.16	3604.16	3604.16	3604.16
**# indels per 100 kbp**	23.77	23.77	23.77	23.77	23.77
**Largest alignment**	63410	63410	63410	63410	63410
**Total aligned length**	988081	988081	988081	988081	988081
**NGA50**	-	-	-	-	-

Escherichia coli

Supplementary Table 1-15 Assembly statistics of gap filler on the Escherichia coli dataset. All statistics are based on contigs of size≥500 bp, unless otherwise noted (e.g., "# contigs(≥0 bp)" and "Total length (≥0 bp)" include all contigs).

**Assembly**	**Scaffold**	**GapCloser**	**RFfiller**	**Sealer**	**TGS-GapCloser**
**# contigs (>= 0 bp)**	26	26	26	26	26
**# contigs (>= 1000 bp)**	26	26	26	26	26
**# contigs (>= 5000 bp)**	23	23	23	23	23
**# contigs (>= 10000 bp)**	21	21	21	21	21
**# contigs (>= 25000 bp)**	18	18	18	18	18
**# contigs (>= 50000 bp)**	13	13	13	13	13
**Total length (>= 0 bp)**	6070239	6070239	6070239	6070239	6070239
**Total length (>= 1000 bp)**	6070239	6070239	6070239	6070239	6070239
**Total length (>= 5000 bp)**	6062523	6062523	6062523	6062523	6062523
**Total length (>= 10000 bp)**	6048809	6048809	6048809	6048809	6048809
**Total length (>= 25000 bp)**	6007231	6007231	6007231	6007231	6007231
**Total length (>= 50000 bp)**	5814405	5814405	5814405	5814405	5814405
**# contigs**	26	26	26	26	26

**Largest contig**	1406276	1406276	1406276	1406276	1406276
**Total length**	6070239	6070239	6070239	6070239	6070239
**Reference length**	4641652	4641652	4641652	4641652	4641652
**GC (%)**	50.65	50.65	50.65	50.65	50.65
**Reference GC (%)**	50.79	50.79	50.79	50.79	50.79
**N50**	1064014	1064014	1064014	1064014	1064014
**NG50**	1349250	1349250	1349250	1349250	1349250
**N75**	384337	384337	384337	384337	384337
**NG75**	1064014	1064014	1064014	1064014	1064014
**L50**	3	3	3	3	3
**LG50**	2	2	2	2	2
**L75**	5	5	5	5	5
**LG75**	3	3	3	3	3
**# misassemblies**	182	182	182	182	182
**# misassembled contigs**	7	7	7	7	7
**Misassembled contigs length**	5231896	5231896	5231896	5231896	5231896
**# local misassemblies**	197	197	197	197	197
**# scaffold gap ext. mis.**	0	0	0	0	0
**# scaffold gap loc. mis.**	0	0	0	0	0
**# unaligned mis. contigs**	7	7	7	7	7
**# unaligned contigs**	9 + 17 part	9 + 17 part	9 + 17 part	9 + 17 part	9 + 17 part
**Unaligned length**	1862024	1862024	1862024	1862024	1862024
**Genome fraction (%)**	89.532	89.532	89.532	89.532	89.532
**Duplication ratio**	1.013	1.013	1.013	1.013	1.013
**# N's per 100 kbp**	0	0	0	0	0
**# mismatches per 100 kbp**	2234.52	2234.52	2234.52	2234.52	2234.52
**# indels per 100 kbp**	31.11	31.11	31.11	31.11	31.11
**Largest alignment**	146930	146930	146930	146930	146930
**Total aligned length**	4204188	4204188	4204188	4204188	4204188
**NA50**	27470	27470	27470	27470	27470

**NGA50**	51984	51984	51984	51984	51984
**NGA75**	18280	18280	18280	18280	18280
**LA50**	51	51	51	51	51
**LGA50**	31	31	31	31	31
**LGA75**	70	70	70	70	70

Homo sapien

Supplementary Table 1-16 Assembly statistics of gap filler on the Homo sapien dataset. All statistics are based on contigs of size≥500 bp, unless otherwise noted (e.g., "# contigs(≥0 bp)" and "Total length (≥0 bp)" include all contigs).

**Assembly**	**Scaffold**	**GapCloser**	**RFfiller**	**Sealer**	**TGS-GapCloser**
**# contigs (>= 0 bp)**	1619	1619	1619	1619	1619
**# contigs (>= 1000 bp)**	1591	1591	1591	1591	1591
**# contigs (>= 5000 bp)**	1525	1525	1525	1525	1525
**# contigs (>= 10000 bp)**	1430	1430	1430	1430	1430
**# contigs (>= 25000 bp)**	1118	1118	1118	1118	1118
**# contigs (>= 50000 bp)**	247	247	247	247	247
**Total length (>= 0 bp)**	55673606	55673606	55673606	55673606	55673606
**Total length (>= 1000 bp)**	55653445	55653445	55653445	55653445	55653445
**Total length (>= 5000 bp)**	55478785	55478785	55478785	55478785	55478785
**Total length (>= 10000 bp)**	54776654	54776654	54776654	54776654	54776654
**Total length (>= 25000 bp)**	48917431	48917431	48917431	48917431	48917431
**Total length (>= 50000 bp)**	17890925	17890925	17890925	17890925	17890925
**# contigs**	1617	1617	1617	1617	1617
**Largest contig**	248991	248991	248991	248991	248991
**Total length**	55673067	55673067	55673067	55673067	55673067
**Reference length**	3272089205	3272089205	3272089205	3272089205	3272089205
**GC (%)**	40.12	40.12	40.12	40.12	40.12
**Reference GC (%)**	41.04	41.04	41.04	41.04	41.04

**N50**	40853	40853	40853	40853	40853
**N75**	30918	30918	30918	30918	30918
**L50**	469	469	469	469	469
**L75**	862	862	862	862	862
**# misassemblies**	749	749	749	749	749
**# misassembled contigs**	396	396	396	396	396
**Misassembled contigs length**	18676839	18676839	18676839	18676839	18676839
**# local misassemblies**	1689	1689	1689	1689	1689
**# scaffold gap ext. mis.**	0	0	0	0	0
**# scaffold gap loc. mis.**	0	0	0	0	0
**# unaligned mis. contigs**	45	45	45	45	45
**# unaligned contigs**	15 + 343 part	15 + 343 part	15 + 343 part	15 + 343 part	15 + 343 part
**Unaligned length**	2005869	2005869	2005869	2005869	2005869
**Genome fraction (%)**	1.713	1.713	1.713	1.713	1.713
**Duplication ratio**	1.007	1.007	1.007	1.007	1.007
**# N's per 100 kbp**	0	0	0	0	0
**# mismatches per 100 kbp**	1295.24	1295.24	1295.24	1295.24	1295.24
**# indels per 100 kbp**	338.43	338.43	338.43	338.43	338.43
**Largest alignment**	147406	147406	147406	147406	147406
**Total aligned length**	53251209	53251209	53251209	53251209	53251209
**NA50**	33508	33508	33508	33508	33508
**NGA50**	-	-	-	-	-
**NA75**	24217	24217	24217	24217	24217
**LA50**	587	587	587	587	587
**LA75**	1070	1070	1070	1070	1070

Saccharomyces cerevisiae

Supplementary Table 1-17 Assembly statistics of gap filler on the Saccharomyces cerevisiae dataset. All statistics are based on contigs of size≥500 bp, unless otherwise noted (e.g., "# contigs(≥0 bp)" and "Total length (≥0 bp)" include all contigs).

Assembly	Scaffold	GapCloser	RFfiller	Sealer	TGS-GapCloser
**# contigs (>= 0 bp)**	1832	1832	1832	1832	1832
**# contigs (>= 1000 bp)**	1797	1797	1797	1797	1797
**# contigs (>= 5000 bp)**	1754	1753	1754	1754	1754
**# contigs (>= 10000 bp)**	1666	1667	1666	1666	1666
**# contigs (>= 25000 bp)**	1421	1424	1421	1421	1421
**# contigs (>= 50000 bp)**	844	846	844	844	844
**Total length (>= 0 bp)**	105678645	105581485	105678645	105678645	105678645
**Total length (>= 1000 bp)**	105656791	105557593	105656791	105656791	105656791
**Total length (>= 5000 bp)**	105521375	105421149	105521375	105521375	105521375
**Total length (>= 10000 bp)**	104837070	104748508	104837070	104837070	104837070
**Total length (>= 25000 bp)**	100631269	100581083	100631269	100631269	100631269
**Total length (>= 50000 bp)**	78529145	78560947	78529145	78529145	78529145
**# contigs**	1831	1832	1831	1831	1831
**Largest contig**	404821	404821	404821	404821	404821
**Total length**	105678154	105581485	105678154	105678154	105678154
**Reference length**	12157105	12157105	12157105	12157105	12157105
**GC (%)**	37.24	37.24	37.24	37.24	37.24
**Reference GC (%)**	38.15	38.15	38.15	38.15	38.15
**N50**	77564	77191	77564	77564	77564
**NG50**	211569	214581	211569	211569	211569
**N75**	49374	49468	49374	49374	49374
**NG75**	184263	184269	184263	184263	184263
**L50**	432	432	432	432	432
**LG50**	24	24	24	24	24
**L75**	859	859	859	859	859
**LG75**	39	39	39	39	39

**# misassemblies**	0	0	0	0	0
**# misassembled contigs**	0	0	0	0	0
**Misassembled contigs length**	0	0	0	0	0
**# local misassemblies**	0	0	0	0	0
**# scaffold gap ext. mis.**	0	0	0	0	0
**# scaffold gap loc. mis.**	0	0	0	0	0
**# unaligned mis. contigs**	0	0	0	0	0
**# unaligned contigs**	1826 + 4 part	1828 + 4 part	1826 + 4 part	1826 + 4 part	1826 + 4 part
**Unaligned length**	105676901	105581016	105676901	105676901	105676901
**Genome fraction (%)**	0.003	0.001	0.003	0.003	0.003
**Duplication ratio**	3.132	3.374	3.132	3.132	3.132
**# N's per 100 kbp**	0.28	0.19	0.28	0.28	0.28
**# mismatches per 100 kbp**	3500	719.42	3500	3500	3500
**# indels per 100 kbp**	0	0	0	0	0
**Largest alignment**	262	72	262	262	262
**Total aligned length**	531	269	531	531	531
**NGA50**	-	-	-	-	-

Staphylococcus aureus

Supplementary Table 1-18 Assembly statistics of gap filler on the Staphylococcus aureus dataset. All statistics are based on contigs of size≥500 bp, unless otherwise noted (e.g., "# contigs(≥0 bp)" and "Total length (≥0 bp)" include all contigs).

**Assembly**	**Scaffold**	**GapCloser**	**RFfiller**	**Sealer**	**TGS-GapCloser**
**# contigs (>= 0 bp)**	1	1	1	1	1
**# contigs (>= 1000 bp)**	1	1	1	1	1
**# contigs (>= 5000 bp)**	1	1	1	1	1
**# contigs (>= 10000 bp)**	1	1	1	1	1
**# contigs (>= 25000 bp)**	1	1	1	1	1
**# contigs (>= 50000 bp)**	1	1	1	1	1

**Total length (>= 0 bp)**	2896521	2896521	2896521	2896521	2896521
**Total length (>= 1000 bp)**	2896521	2896521	2896521	2896521	2896521
**Total length (>= 5000 bp)**	2896521	2896521	2896521	2896521	2896521
**Total length (>= 10000 bp)**	2896521	2896521	2896521	2896521	2896521
**Total length (>= 25000 bp)**	2896521	2896521	2896521	2896521	2896521
**Total length (>= 50000 bp)**	2896521	2896521	2896521	2896521	2896521
**# contigs**	1	1	1	1	1
**Largest contig**	2896521	2896521	2896521	2896521	2896521
**Total length**	2896521	2896521	2896521	2896521	2896521
**Reference length**	2821361	2821361	2821361	2821361	2821361
**GC (%)**	32.74	32.74	32.74	32.74	32.74
**Reference GC (%)**	32.87	32.87	32.87	32.87	32.87
**N50**	2896521	2896521	2896521	2896521	2896521
**NG50**	2896521	2896521	2896521	2896521	2896521
**N75**	2896521	2896521	2896521	2896521	2896521
**NG75**	2896521	2896521	2896521	2896521	2896521
**L50**	1	1	1	1	1
**LG50**	1	1	1	1	1
**L75**	1	1	1	1	1
**LG75**	1	1	1	1	1
**# misassemblies**	54	54	54	54	54
**# misassembled contigs**	1	1	1	1	1
**Misassembled contigs length**	2896521	2896521	2896521	2896521	2896521
**# local misassemblies**	30	30	30	30	30
**# scaffold gap ext. mis.**	0	0	0	0	0
**# scaffold gap loc. mis.**	0	0	0	0	0
**# unaligned mis. contigs**	0	0	0	0	0
**# unaligned contigs**	0 + 1 part	0 + 1 part	0 + 1 part	0 + 1 part	0 + 1 part
**Unaligned length**	274765	274765	274765	274765	274765
**Genome fraction (%)**	92.509	92.509	92.509	92.509	92.509
**Duplication ratio**	1.004	1.004	1.004	1.004	1.004

**# N's per 100 kbp**	0	0	0	0	0
**# mismatches per 100 kbp**	192.3	192.3	192.3	192.3	192.3
**# indels per 100 kbp**	11.57	11.57	11.57	11.57	11.57
**Largest alignment**	329938	329938	329938	329938	329938
**Total aligned length**	2617887	2617887	2617887	2617887	2617887
**NA50**	92541	92541	92541	92541	92541
**NGA50**	92541	92541	92541	92541	92541
**NA75**	54665	54665	54665	54665	54665
**NGA75**	56975	56975	56975	56975	56975
**LA50**	8	8	8	8	8
**LGA50**	8	8	8	8	8
**LA75**	19	19	19	19	19
**LGA75**	18	18	18	18	18

SGA

Arabidopsis thaliana

Supplementary Table 1-19 Assembly statistics of gap filler on the Arabidopsis thaliana dataset. All statistics are based on contigs of size≥500 bp, unless otherwise noted (e.g., "# contigs(≥0 bp)" and "Total length (≥0 bp)" include all contigs).

**Assembly**	**Scaffold**	**GapCloser**	**RFfiller**	**Sealer**	**TGS-GapCloser**
**# contigs (>= 0 bp)**	25890	25890	25890	25890	25890
**# contigs (>= 1000 bp)**	101	101	101	101	101
**# contigs (>= 5000 bp)**	0	0	0	0	0
**# contigs (>= 10000 bp)**	0	0	0	0	0
**# contigs (>= 25000 bp)**	0	0	0	0	0
**# contigs (>= 50000 bp)**	0	0	0	0	0
**Total length (>= 0 bp)**	5516863	5516863	5516863	5516863	5516863
**Total length (>= 1000 bp)**	146311	146311	146311	146311	146311
**Total length (>= 5000 bp)**	0	0	0	0	0

**Total length (>= 10000 bp)**	0	0	0	0	0
**Total length (>= 25000 bp)**	0	0	0	0	0
**Total length (>= 50000 bp)**	0	0	0	0	0
**# contigs**	416	416	416	416	416
**Largest contig**	3712	3712	3712	3712	3712
**Total length**	363178	363178	363178	363178	363178
**Reference length**	119668634	119668634	119668634	119668634	119668634
**GC (%)**	41.78	41.78	41.78	41.78	41.78
**Reference GC (%)**	36.06	36.06	36.06	36.06	36.06
**N50**	886	886	886	886	886
**N75**	665	665	665	665	665
**L50**	139	139	139	139	139
**L75**	258	258	258	258	258
**# misassemblies**	0	0	0	0	0
**# misassembled contigs**	0	0	0	0	0
**Misassembled contigs length**	0	0	0	0	0
**# local misassemblies**	2	2	2	2	2
**# scaffold gap ext. mis.**	0	0	0	0	0
**# scaffold gap loc. mis.**	0	0	0	0	0
**# unaligned mis. contigs**	1	1	1	1	1
**# unaligned contigs**	4 + 0 part	4 + 0 part	4 + 0 part	4 + 0 part	4 + 0 part
**Unaligned length**	2407	2407	2407	2407	2407
**Genome fraction (%)**	0.296	0.296	0.296	0.296	0.296
**Duplication ratio**	1.02	1.02	1.02	1.02	1.02
**# N's per 100 kbp**	0	0	0	0	0
**# mismatches per 100 kbp**	78.6	78.6	78.6	78.6	78.6
**# indels per 100 kbp**	2.83	2.83	2.83	2.83	2.83
**Largest alignment**	3712	3712	3712	3712	3712
**Total aligned length**	359572	359572	359572	359572	359572
**NA50**	886	886	886	886	886
**NGA50**	-	-	-	-	-

**NA75**	662	662	662	662	662
**LA50**	139	139	139	139	139
**LA75**	258	258	258	258	258

Bacillus cereus

Supplementary Table 1-20 Assembly statistics of gap filler on the Bacillus cereus dataset. All statistics are based on contigs of size≥500 bp, unless otherwise noted (e.g., "# contigs(≥0 bp)" and "Total length (≥0 bp)" include all contigs).

**Assembly**	**Scaffold**	**GapCloser**	**RFfiller**	**Sealer**	**TGS-GapCloser**
**# contigs (>= 0 bp)**	13259	13259	13259	13259	13259
**# contigs (>= 1000 bp)**	742	742	742	742	742
**# contigs (>= 5000 bp)**	346	346	346	346	346
**# contigs (>= 10000 bp)**	154	154	154	154	154
**# contigs (>= 25000 bp)**	24	24	24	24	23
**# contigs (>= 50000 bp)**	4	4	4	4	4
**Total length (>= 0 bp)**	9166123	9166159	9166123	9166123	9164585
**Total length (>= 1000 bp)**	5251702	5251738	5251702	5251702	5250164
**Total length (>= 5000 bp)**	4252327	4252360	4252327	4252327	4250789
**Total length (>= 10000 bp)**	2896671	2896693	2896671	2896671	2895133
**Total length (>= 25000 bp)**	947293	947305	947293	947293	921306
**Total length (>= 50000 bp)**	245882	245886	245882	245882	245231
**# contigs**	1019	1019	1019	1019	1019
**Largest contig**	72911	72911	72911	72911	72911
**Total length**	5426893	5426929	5426893	5426893	5425355
**Reference length**	5427083	5427083	5427083	5427083	5427083
**GC (%)**	35.52	35.52	35.52	35.52	35.52
**Reference GC (%)**	35.29	35.29	35.29	35.29	35.29
**N50**	10734	10734	10734	10734	10734

**NG50**	10734	10734	10734	10734	10734
**N75**	5551	5551	5551	5551	5551
**NG75**	5551	5551	5551	5551	5551
**L50**	137	137	137	137	137
**LG50**	137	137	137	137	137
**L75**	312	312	312	312	312
**LG75**	312	312	312	312	312
**# misassemblies**	1	1	1	1	1
**# misassembled contigs**	1	1	1	1	1
**Misassembled contigs length**	502	502	502	502	502
**# local misassemblies**	9	9	9	9	9
**# scaffold gap ext. mis.**	0	0	0	0	0
**# scaffold gap loc. mis.**	0	0	0	0	0
**# unaligned mis. contigs**	107	107	107	107	107
**# unaligned contigs**	680 + 267 part	680 + 267 part	680 + 267 part	680 + 267 part	680 + 267 part
**Unaligned length**	5078526	5079487	5078576	5078526	5076131
**Genome fraction (%)**	6.04	6.039	6.04	6.04	6.063
**Duplication ratio**	1.063	1.06	1.063	1.063	1.061
**# N's per 100 kbp**	16.58	0.66	15.66	16.58	13.36
**# mismatches per 100 kbp**	3808.86	3810.47	3808.86	3808.86	3811.37
**# indels per 100 kbp**	46.37	46.38	46.37	46.37	46.19
**Largest alignment**	10096	10096	10096	10096	10096
**Total aligned length**	342625	342564	342625	342625	343657
**NGA50**	-	-	-	-	-

Escherichia coli

Supplementary Table 1-21 Assembly statistics of gap filler on the Escherichia coli dataset. All statistics are based on contigs of size≥500 bp, unless otherwise noted (e.g., ”# contigs(≥0 bp)” and ”Total length (≥0 bp)” include all contigs).

Assembly	Scaffold	GapCloser	RFfiller	Sealer	TGS-GapCloser
# contigs (>= 0 bp)	7939	7939	7939	7939	7939
# contigs (>= 1000 bp)	988	988	988	988	988
# contigs (>= 5000 bp)	365	365	365	365	365
# contigs (>= 10000 bp)	138	138	138	138	138
# contigs (>= 25000 bp)	12	12	12	12	12
# contigs (>= 50000 bp)	0	0	0	0	0
Total length (>= 0 bp)	7474211	7474211	7474211	7474211	7474211
Total length (>= 1000 bp)	5327907	5327907	5327907	5327907	5327907
Total length (>= 5000 bp)	3731827	3731827	3731827	3731827	3731827
Total length (>= 10000 bp)	2140162	2140162	2140162	2140162	2140162
Total length (>= 25000 bp)	352297	352297	352297	352297	352297
Total length (>= 50000 bp)	0	0	0	0	0
# contigs	1304	1304	1304	1304	1304
Largest contig	42367	42367	42367	42367	42367
Total length	5549396	5549396	5549396	5549396	5549396
Reference length	4641652	4641652	4641652	4641652	4641652
GC (%)	50.27	50.27	50.27	50.27	50.27
Reference GC (%)	50.79	50.79	50.79	50.79	50.79
N50	7681	7681	7681	7681	7681
NG50	9358	9358	9358	9358	9358
N75	3985	3985	3985	3985	3985
NG75	5595	5595	5595	5595	5595
L50	211	211	211	211	211
LG50	157	157	157	157	157
L75	461	461	461	461	461

**LG75**	318	318	318	318	318
**# misassemblies**	52	52	52	52	52
**# misassembled contigs**	51	51	51	51	51
**Misassembled contigs length**	575881	575881	575881	575881	575881
**# local misassemblies**	126	126	126	126	126
**# scaffold gap ext. mis.**	0	0	0	0	0
**# scaffold gap loc. mis.**	0	0	0	0	0
**# unaligned mis. contigs**	20	20	20	20	20
**# unaligned contigs**	407 + 190 part	407 + 190 part	407 + 190 part	407 + 190 part	407 + 190 part
**Unaligned length**	1332309	1332309	1332309	1332309	1332309
**Genome fraction (%)**	87.129	87.129	87.129	87.129	87.129
**Duplication ratio**	1.043	1.043	1.043	1.043	1.043
**# N's per 100 kbp**	0	0	0	0	0
**# mismatches per 100 kbp**	1681.48	1681.48	1681.48	1681.48	1681.48
**# indels per 100 kbp**	21.69	21.69	21.69	21.69	21.69
**Largest alignment**	41457	41457	41457	41457	41457
**Total aligned length**	4193611	4193611	4193611	4193611	4193611
**NA50**	5205	5205	5205	5205	5205
**NGA50**	6669	6669	6669	6669	6669
**NA75**	543	543	543	543	543
**NGA75**	3130	3130	3130	3130	3130
**LA50**	278	278	278	278	278
**LGA50**	200	200	200	200	200
**LA75**	873	873	873	873	873
**LGA75**	450	450	450	450	450

Homo sapien

Supplementary Table 1-22 Assembly statistics of gap filler on the Homo sapien dataset. All statistics are based on contigs of size≥500 bp, unless otherwise noted (e.g., "# contigs(≥0 bp)" and "Total length (≥0 bp)" include all contigs).

Assembly	Scaffold	GapCloser	RFfiller	Sealer	TGS-GapCloser
**# contigs (>= 0 bp)**	72325	72325	72325	72325	72325
**# contigs (>= 1000 bp)**	89	89	89	89	89
**# contigs (>= 5000 bp)**	9	9	9	9	9
**# contigs (>= 10000 bp)**	2	2	2	2	2
**# contigs (>= 25000 bp)**	0	0	0	0	0
**# contigs (>= 50000 bp)**	0	0	0	0	0
**Total length (>= 0 bp)**	9282063	9282063	9282063	9282063	9282063
**Total length (>= 1000 bp)**	221123	221123	221123	221123	221123
**Total length (>= 5000 bp)**	73666	73666	73666	73666	73666
**Total length (>= 10000 bp)**	27529	27529	27529	27529	27529
**Total length (>= 25000 bp)**	0	0	0	0	0
**Total length (>= 50000 bp)**	0	0	0	0	0
**# contigs**	207	207	207	207	207
**Largest contig**	16670	16670	16670	16670	16670
**Total length**	300785	300785	300785	300785	300785
**Reference length**	3272089205	3272089205	3272089205	3272089205	3272089205
**GC (%)**	50.89	50.89	50.89	50.89	50.89
**Reference GC (%)**	41.04	41.04	41.04	41.04	41.04
**N50**	1829	1829	1829	1829	1829
**N75**	958	958	958	958	958
**L50**	37	37	37	37	37
**L75**	94	94	94	94	94
**# misassemblies**	0	0	0	0	0
**# misassembled contigs**	0	0	0	0	0
**Misassembled contigs length**	0	0	0	0	0
**# local misassemblies**	2	2	2	2	2
**# scaffold gap ext. mis.**	0	0	0	0	0

**# scaffold gap loc. mis.**	0	0	0	0	0
**# unaligned mis. contigs**	4	4	4	4	4
**# unaligned contigs**	67 + 3 part	67 + 3 part	67 + 3 part	67 + 3 part	67 + 3 part
**Unaligned length**	144523	144523	144523	144523	144523
**Genome fraction (%)**	0.005	0.005	0.005	0.005	0.005
**Duplication ratio**	1.014	1.014	1.014	1.014	1.014
**# N's per 100 kbp**	0	0	0	0	0
**# mismatches per 100 kbp**	456.22	456.22	456.22	456.22	456.22
**# indels per 100 kbp**	22.06	22.06	22.06	22.06	22.06
**Largest alignment**	16670	16670	16670	16670	16670
**Total aligned length**	154701	154701	154701	154701	154701
**NA50**	516	516	516	516	516
**NGA50**	-	-	-	-	-
**LA50**	129	129	129	129	129

Saccharomyces cerevisiae

Supplementary Table 1-23 Assembly statistics of gap filler on the Saccharomyces cerevisiae dataset. All statistics are based on contigs of size≥500 bp, unless otherwise noted (e.g., "# contigs(≥0 bp)" and "Total length (≥0 bp)" include all contigs).

**Assembly**	**Scaffold**	**GapCloser**	**RFfiller**	**Sealer**	**TGS-GapCloser**
**# contigs (>= 0 bp)**	2857	2857	2857	2857	2857
**# contigs (>= 1000 bp)**	188	188	188	188	188
**# contigs (>= 5000 bp)**	101	101	101	101	102
**# contigs (>= 10000 bp)**	89	89	89	89	89
**# contigs (>= 25000 bp)**	73	73	73	73	73
**# contigs (>= 50000 bp)**	60	60	60	60	60
**Total length (>= 0 bp)**	11954175	11954999	11954175	11953804	11975674
**Total length (>= 1000 bp)**	11513019	11513837	11513019	11512652	11534518
**Total length (>= 5000 bp)**	11314988	11315801	11314988	11314503	11340701
**Total length (>= 10000 bp)**	11226082	11226894	11226082	11225597	11241969

**Total length (>= 25000 bp)**	10978133	10978946	10978133	10977705	10994082
**Total length (>= 50000 bp)**	10479239	10480100	10479239	10478747	10494004
**# contigs**	266	266	266	267	266
**Largest contig**	716641	716598	716641	716578	716353
**Total length**	11569315	11570136	11569315	11569539	11590814
**Reference length**	12157105	12157105	12157105	12157105	12157105
**GC (%)**	38.1	38.09	38.1	38.1	38.1
**Reference GC (%)**	38.15	38.15	38.15	38.15	38.15
**N50**	202666	202938	202666	202564	202485
**NG50**	192327	192533	192327	192314	192337
**N75**	114862	114863	114862	114862	114862
**NG75**	109169	109169	109169	109169	109169
**L50**	20	20	20	20	20
**LG50**	21	21	21	21	21
**L75**	39	39	39	39	39
**LG75**	43	43	43	43	43
**# misassemblies**	70	83	71	68	79
**# misassembled contigs**	48	50	46	46	51
**Misassembled contigs length**	5891283	6304036	5728098	5728217	6058934
**# local misassemblies**	96	134	131	105	113
**# scaffold gap ext. mis.**	10	0	6	9	4
**# scaffold gap loc. mis.**	58	1	23	36	23
**# unaligned mis. contigs**	7	9	8	8	9
**# unaligned contigs**	9 + 34 part	9 + 34 part	9 + 35 part	9 + 33 part	9 + 38 part
**Unaligned length**	103273	102044	106054	102386	129380
**Genome fraction (%)**	93.849	93.924	93.849	93.88	93.885
**Duplication ratio**	1.005	1.004	1.005	1.005	1.004
**# N's per 100 kbp**	124.26	4	63.1	77.82	45.43
**# mismatches per 100 kbp**	582.01	586.02	582.73	583.82	585.39
**# indels per 100 kbp**	50.4	50.72	50.94	50.47	53.62

**Largest alignment**	529154	529230	529154	529105	529030
**Total aligned length**	11428586	11440151	11428604	11432292	11431473
**NA50**	124209	123845	124209	123845	123088
**NGA50**	121483	121483	121483	118186	114505
**NA75**	73219	66746	69933	70254	69004
**NGA75**	62193	58874	60394	62193	60394
**LA50**	27	28	27	27	28
**LGA50**	30	30	30	30	31
**LA75**	56	59	57	57	59
**LGA75**	62	66	64	63	65

Staphylococcus aureus

Supplementary Table 1-24 Assembly statistics of gap filler on the Staphylococcus aureus dataset. All statistics are based on contigs of size≥500 bp, unless otherwise noted (e.g., ”# contigs(≥0 bp)” and ”Total length (≥0 bp)” include all contigs).

**Assembly**	**Scaffold**	**GapCloser**	**RFfiller**	**Sealer**	**TGS-GapCloser**
**# contigs (>= 0 bp)**	2151	2151	2151	2151	2151
**# contigs (>= 1000 bp)**	107	107	107	107	106
**# contigs (>= 5000 bp)**	74	74	74	74	74
**# contigs (>= 10000 bp)**	60	60	60	60	60
**# contigs (>= 25000 bp)**	44	44	44	44	45
**# contigs (>= 50000 bp)**	20	20	20	20	20
**Total length (>= 0 bp)**	3483776	3485701	3483776	3485160	3467274
**Total length (>= 1000 bp)**	2809568	2811478	2809568	2810952	2792349
**Total length (>= 5000 bp)**	2730340	2732222	2730340	2731724	2715751
**Total length (>= 10000 bp)**	2623567	2625435	2623567	2624951	2609842
**Total length (>= 25000 bp)**	2341652	2341779	2341652	2341652	2355057
**Total length (>= 50000 bp)**	1468332	1468409	1468332	1468332	1464271
**# contigs**	151	151	151	151	151
**Largest contig**	161938	161942	161938	161938	163105

**Total length**	2838469	2840394	2838469	2839853	2821967
**Reference length**	2821361	2821361	2821361	2821361	2821361
**GC (%)**	32.64	32.65	32.64	32.64	32.65
**Reference GC (%)**	32.87	32.87	32.87	32.87	32.87
**N50**	52175	52176	52175	52175	51888
**NG50**	52333	52335	52333	52333	51888
**N75**	32618	32618	32618	32618	32618
**NG75**	32618	32618	32618	32618	32618
**L50**	20	20	20	20	19
**LG50**	19	19	19	19	19
**L75**	37	37	37	37	37
**LG75**	37	37	37	37	37
**# misassemblies**	51	54	51	51	50
**# misassembled contigs**	33	33	33	33	33
**Misassembled contigs length**	1207143	1258858	1207143	1207143	1105863
**# local misassemblies**	75	228	124	75	79
**# scaffold gap ext. mis.**	5	0	4	5	4
**# scaffold gap loc. mis.**	151	0	102	151	34
**# unaligned mis. contigs**	4	4	4	4	4
**# unaligned contigs**	34 + 47 part	34 + 47 part	34 + 48 part	34 + 47 part	34 + 47 part
**Unaligned length**	300396	303303	302204	301872	292966
**Genome fraction (%)**	87.818	87.823	87.814	87.818	88.356
**Duplication ratio**	1.024	1.024	1.024	1.024	1.015
**# N's per 100 kbp**	183.2	7.15	121.54	179.87	69.99
**# mismatches per 100 kbp**	1361.12	1363.59	1361.03	1361.12	1389.62
**# indels per 100 kbp**	45.73	45.85	45.73	45.73	55.6
**Largest alignment**	107334	107430	107334	107334	160661
**Total aligned length**	2521599	2525370	2521510	2521599	2515950
**NA50**	35516	35541	35519	35516	36053
**NGA50**	35516	35541	35519	35516	36053

**NA75**	15402	15402	15402	15402	15602
**NGA75**	15602	15602	15602	15602	15602
**LA50**	26	26	26	26	25
**LGA50**	26	26	26	26	25
**LA75**	55	55	55	55	53
**LGA75**	54	54	54	54	53

SOAPdenovo2

Arabidopsis thaliana

Supplementary Table 1-25 Assembly statistics of gap filler on the Arabidopsis thaliana dataset. All statistics are based on contigs of size≥500 bp, unless otherwise noted (e.g., ”# contigs(≥0 bp)” and ”Total length (≥0 bp)” include all contigs).

**Assembly**	**Scaffold**	**GapCloser**	**RFfiller**	**Sealer**	**TGS-GapCloser**
**# contigs (>= 0 bp)**	12890	12890	12890	12890	12890
**# contigs (>= 1000 bp)**	90	90	90	91	90
**# contigs (>= 5000 bp)**	0	0	0	0	0
**# contigs (>= 10000 bp)**	0	0	0	0	0
**# contigs (>= 25000 bp)**	0	0	0	0	0
**# contigs (>= 50000 bp)**	0	0	0	0	0
**Total length (>= 0 bp)**	2607468	2607890	2607468	2609331	2607724
**Total length (>= 1000 bp)**	137955	138133	137955	139632	138078
**Total length (>= 5000 bp)**	0	0	0	0	0
**Total length (>= 10000 bp)**	0	0	0	0	0
**Total length (>= 25000 bp)**	0	0	0	0	0
**Total length (>= 50000 bp)**	0	0	0	0	0
**# contigs**	331	331	331	334	331
**Largest contig**	4241	4237	4241	4237	4241
**Total length**	302457	302883	302457	305687	302713
**Reference length**	119668634	119668634	119668634	119668634	119668634

**GC (%)**	40.67	40.71	40.68	40.71	40.69
**Reference GC (%)**	36.06	36.06	36.06	36.06	36.06
**N50**	923	924	923	929	928
**N75**	672	672	672	673	672
**L50**	104	104	104	105	104
**L75**	201	201	201	202	201
**# misassemblies**	8	5	6	4	8
**# misassembled contigs**	8	5	6	4	8
**Misassembled contigs length**	5101	3527	3994	2906	5101
**# local misassemblies**	3	3	3	0	2
**# scaffold gap ext. mis.**	0	0	0	0	0
**# scaffold gap loc. mis.**	3	0	3	0	3
**# unaligned mis. contigs**	0	0	0	0	0
**# unaligned contigs**	2 + 0 part	1 + 0 part	2 + 0 part	2 + 0 part	2 + 0 part
**Unaligned length**	1113	577	1187	1156	1113
**Genome fraction (%)**	0.25	0.251	0.25	0.253	0.25
**Duplication ratio**	1.009	1.006	1.008	1.006	1.008
**# N's per 100 kbp**	1037.17	65.7	761.43	84.07	739.97
**# mismatches per 100 kbp**	101.79	106.5	154.58	108.31	124.03
**# indels per 100 kbp**	42.52	31.95	58.89	7.26	76.22
**Largest alignment**	4241	4237	4241	4237	4241
**Total aligned length**	299308	300945	299515	303865	299936
**NA50**	918	924	918	925	918
**NGA50**	-	-	-	-	-
**NA75**	664	669	664	670	667
**LA50**	105	104	105	105	105
**LA75**	203	202	203	203	202

Bacillus cereus

Supplementary Table 1-26 Assembly statistics of gap filler on the Bacillus cereus dataset. All statistics are based on contigs of size≥500 bp, unless otherwise noted (e.g., "# contigs(≥0 bp)" and "Total length (≥0 bp)" include all contigs).

Assembly	Scaffold	GapCloser	RFfiller	Sealer	TGS-GapCloser
# contigs (>= 0 bp)	3092	3092	3092	3092	3092
# contigs (>= 1000 bp)	1179	1183	1179	1188	1179
# contigs (>= 5000 bp)	269	269	269	269	269
# contigs (>= 10000 bp)	73	73	73	73	73
# contigs (>= 25000 bp)	6	6	6	6	6
# contigs (>= 50000 bp)	0	0	0	0	0
Total length (>= 0 bp)	5262278	5267911	5262278	5277065	5262921
Total length (>= 1000 bp)	4527195	4535619	4527195	4545222	4527838
Total length (>= 5000 bp)	2507817	2509247	2507817	2510132	2507743
Total length (>= 10000 bp)	1153105	1153356	1153105	1153455	1153035
Total length (>= 25000 bp)	195984	195985	195984	196025	195984
Total length (>= 50000 bp)	0	0	0	0	0
# contigs	1701	1702	1701	1709	1701
Largest contig	49021	49021	49021	49021	49021
Total length	4903642	4909719	4903642	4920745	4904285
Reference length	5427083	5427083	5427083	5427083	5427083
GC (%)	35.8	35.81	35.8	35.81	35.8
Reference GC (%)	35.29	35.29	35.29	35.29	35.29
N50	5084	5083	5084	5063	5084
NG50	4353	4371	4353	4381	4353
N75	2284	2284	2284	2301	2284
NG75	1648	1659	1648	1663	1648
L50	258	259	258	260	258
LG50	313	313	313	313	313
L75	623	624	623	626	623

**LG75**	825	823	825	821	825
**# misassemblies**	1	0	1	0	1
**# misassembled contigs**	1	0	1	0	1
**Misassembled contigs length**	505	0	505	0	505
**# local misassemblies**	6	6	6	7	6
**# scaffold gap ext. mis.**	0	0	0	0	0
**# scaffold gap loc. mis.**	1	0	0	0	1
**# unaligned mis. contigs**	91	92	93	88	91
**# unaligned contigs**	1354 + 291 part	1356 + 289 part	1354 + 291 part	1362 + 288 part	1354 + 291 part
**Unaligned length**	4564742	4575222	4565326	4586409	4565455
**Genome fraction (%)**	5.915	5.93	5.917	5.919	5.915
**Duplication ratio**	1.056	1.039	1.054	1.041	1.056
**# N's per 100 kbp**	106.47	3.89	90.44	7.4	104.68
**# mismatches per 100 kbp**	3883.98	3879.34	3882.83	3871.39	3884.02
**# indels per 100 kbp**	38.32	38.53	38.31	43.89	38.32
**Largest alignment**	27834	27834	27834	27834	27834
**Total aligned length**	321970	322683	322065	322378	321963
**NGA50**	-	-	-	-	-

Escherichia coli

Supplementary Table 1-27 Assembly statistics of gap filler on the Escherichia coli dataset. All statistics are based on contigs of size≥500 bp, unless otherwise noted (e.g., ”# contigs(≥0 bp)” and ”Total length (≥0 bp)” include all contigs).

**Assembly**	**Scaffold**	**GapCloser**	**RFfiller**	**Sealer**	**TGS-GapCloser**
**# contigs (>= 0 bp)**	2300	2300	2300	2300	2300
**# contigs (>= 1000 bp)**	808	815	808	821	809
**# contigs (>= 5000 bp)**	336	336	336	336	336
**# contigs (>= 10000 bp)**	149	149	149	149	149

**# contigs (>= 25000 bp)**	14	14	14	14	14
**# contigs (>= 50000 bp)**	0	0	0	0	0
**Total length (>= 0 bp)**	5477603	5488461	5477603	5504716	5480825
**Total length (>= 1000 bp)**	5038011	5052436	5038011	5065855	5041960
**Total length (>= 5000 bp)**	3859241	3860942	3859241	3863230	3860237
**Total length (>= 10000 bp)**	2502260	2503543	2502260	2505120	2502858
**Total length (>= 25000 bp)**	468550	468603	468550	468623	468622
**Total length (>= 50000 bp)**	0	0	0	0	0
**# contigs**	1067	1072	1067	1089	1067
**Largest contig**	49366	49366	49366	49366	49366
**Total length**	5222480	5235516	5222480	5256850	5225702
**Reference length**	4641652	4641652	4641652	4641652	4641652
**GC (%)**	50.44	50.45	50.44	50.45	50.45
**Reference GC (%)**	50.79	50.79	50.79	50.79	50.79
**N50**	9632	9632	9632	9615	9632
**NG50**	11020	11020	11020	11020	11020
**N75**	4752	4745	4752	4730	4748
**NG75**	6482	6490	6482	6490	6482
**L50**	161	161	161	162	161
**LG50**	132	132	132	132	132
**L75**	348	350	348	353	349
**LG75**	270	270	270	270	270
**# misassemblies**	41	43	41	42	41
**# misassembled contigs**	41	43	41	42	41
**Misassembled contigs length**	450937	456653	450937	456246	451422
**# local misassemblies**	112	124	121	118	110
**# scaffold gap ext. mis.**	2	0	1	1	2
**# scaffold gap loc. mis.**	15	0	8	3	14

**# unaligned mis. contigs**	10	9	10	11	10
**# unaligned contigs**	408 + 161 part	411 + 164 part	408 + 162 part	425 + 164 part	408 + 161 part
**Unaligned length**	1166672	1177897	1168464	1193495	1169052
**Genome fraction (%)**	86.444	86.556	86.435	86.592	86.458
**Duplication ratio**	1.011	1.01	1.01	1.011	1.011
**# N's per 100 kbp**	81.15	2.58	55.43	8.2	73.58
**# mismatches per 100 kbp**	1607.59	1611.32	1607.39	1615.17	1608.76
**# indels per 100 kbp**	20.86	20.91	21.06	20.1	22.75
**Largest alignment**	47395	47395	47395	47395	47395
**Total aligned length**	4025781	4032246	4025326	4036849	4027136
**NA50**	7154	7141	7154	7141	7172
**NGA50**	8412	8372	8372	8441	8441
**NA75**	1177	1175	1175	1122	1175
**NGA75**	3846	3846	3846	3855	3846
**LA50**	192	193	192	194	192
**LGA50**	155	155	155	155	155
**LA75**	539	544	540	557	540
**LGA75**	358	358	358	357	358

Homo sapien

Supplementary Table 1-28 Assembly statistics of gap filler on the Homo sapien dataset. All statistics are based on contigs of size≥500 bp, unless otherwise noted (e.g., "# contigs(≥0 bp)" and "Total length (≥0 bp)" include all contigs).

**Assembly**	**Scaffold**	**GapCloser**	**RFfiller**	**Sealer**	**TGS-GapCloser**
**# contigs (>= 0 bp)**	4391	4391	4391	4391	4391
**# contigs (>= 1000 bp)**	221	182	221	221	221
**# contigs (>= 5000 bp)**	16	13	16	16	15
**# contigs (>= 10000 bp)**	4	4	4	4	5
**# contigs (>= 25000 bp)**	1	1	1	1	1
**# contigs (>= 50000 bp)**	1	1	1	1	1

**Total length (>= 0 bp)**	1508788	1422177	1508788	1508788	1512562
**Total length (>= 1000 bp)**	556143	471222	556143	556143	559950
**Total length (>= 5000 bp)**	195635	176180	195635	195635	195224
**Total length (>= 10000 bp)**	114947	114715	114947	114947	125922
**Total length (>= 25000 bp)**	59808	59586	59808	59808	59615
**Total length (>= 50000 bp)**	59808	59586	59808	59808	59615
**# contigs**	584	504	584	584	584
**Largest contig**	59808	59586	59808	59808	59615
**Total length**	799383	686273	799383	799383	803157
**Reference length**	3272089205	3272089205	3272089205	3272089205	3272089205
**GC (%)**	46.88	46.66	45.95	46.88	46.86
**Reference GC (%)**	41.04	41.04	41.04	41.04	41.04
**N50**	1623	1560	1623	1623	1639
**N75**	839	832	839	839	841
**L50**	99	80	99	99	98
**L75**	269	229	269	269	268
**# misassemblies**	40	26	43	40	39
**# misassembled contigs**	35	24	41	35	35
**Misassembled contigs length**	38081	22243	57512	38081	38065
**# local misassemblies**	1	20	434	1	1
**# scaffold gap ext. mis.**	4	1	0	4	4
**# scaffold gap loc. mis.**	99	30	13	99	102
**# unaligned mis. contigs**	3	1	100	3	3
**# unaligned contigs**	33 + 0 part	25 + 1 part	32 + 9 part	33 + 0 part	33 + 0 part
**Unaligned length**	141000	141858	155090	141000	141177
**Genome fraction (%)**	0.016	0.017	0.014	0.016	0.016
**Duplication ratio**	1.32	1.056	1.457	1.32	1.313
**# N's per 100 kbp**	21756.4	1342.18	328.5	21756.4	21340.29
**# mismatches per 100 kbp**	639.19	852.61	4784.88	639.19	644.66
**# indels per 100 kbp**	401.12	110.16	255.11	401.12	394.85

**Largest alignment**	16597	16597	16597	16597	16597
**Total aligned length**	566688	522892	442368	566688	570729
**NA50**	764	889	260	764	770
**NGA50**	-	-	-	-	-
**NA75**	-	406	-	-	-
**LA50**	232	189	450	232	229
**LA75**	-	469	-	-	-

Saccharomyces cerevisiae

Supplementary Table 1-29 Assembly statistics of gap filler on the Saccharomyces cerevisiae dataset. All statistics are based on contigs of size≥500 bp, unless otherwise noted (e.g., "# contigs(≥0 bp)" and "Total length (≥0 bp)" include all contigs).

**Assembly**	**Scaffold**	**GapCloser**	**RFfiller**	**Sealer**	**TGS-GapCloser**
**# contigs (>= 0 bp)**	7206	7206	7206	7206	7206
**# contigs (>= 1000 bp)**	165	180	165	179	165
**# contigs (>= 5000 bp)**	98	100	98	99	99
**# contigs (>= 10000 bp)**	88	89	88	89	88
**# contigs (>= 25000 bp)**	72	72	72	72	72
**# contigs (>= 50000 bp)**	61	61	61	61	61
**Total length (>= 0 bp)**	12298672	12368295	12298672	12353414	12371593
**Total length (>= 1000 bp)**	11401679	11482654	11401679	11465293	11474600
**Total length (>= 5000 bp)**	11261974	11318433	11261974	11301821	11339281
**Total length (>= 10000 bp)**	11187108	11241910	11187108	11230523	11259079
**Total length (>= 25000 bp)**	10911555	10954383	10911555	10943446	10983212
**Total length (>= 50000 bp)**	10511076	10552285	10511076	10541209	10581166
**# contigs**	239	239	239	246	239
**Largest contig**	713160	716790	713160	715709	716382
**Total length**	11455304	11524905	11455304	11512277	11528225
**Reference length**	12157105	12157105	12157105	12157105	12157105
**GC (%)**	38.19	38.15	38.19	38.16	38.2

**Reference GC (%)**	38.15	38.15	38.15	38.15	38.15
**N50**	205153	205051	205153	205055	205538
**NG50**	189572	190390	189572	189947	189870
**N75**	112078	112341	112078	112255	112799
**NG75**	102223	103642	102223	103629	108496
**L50**	18	18	18	18	18
**LG50**	20	20	20	20	20
**L75**	38	38	38	38	38
**LG75**	43	42	43	42	42
**# misassemblies**	48	71	55	54	58
**# misassembled contigs**	35	43	37	39	38
**Misassembled contigs length**	5805380	6839057	6337884	6160685	6703923
**# local misassemblies**	164	191	227	145	186
**# scaffold gap ext. mis.**	19	0	10	14	16
**# scaffold gap loc. mis.**	188	0	130	52	140
**# unaligned mis. contigs**	4	2	5	3	4
**# unaligned contigs**	23 + 23 part	22 + 27 part	23 + 28 part	24 + 22 part	23 + 32 part
**Unaligned length**	104446	109862	110764	106586	147144
**Genome fraction (%)**	92.727	93.282	92.74	93.181	92.839
**Duplication ratio**	1.007	1.007	1.006	1.007	1.008
**# N's per 100 kbp**	358.43	4.76	226.65	98.66	314.97
**# mismatches per 100 kbp**	565.24	575.06	569.02	574.14	577.45
**# indels per 100 kbp**	59.03	49.96	60.62	49.89	80.23
**Largest alignment**	525730	529282	525738	528547	527938
**Total aligned length**	11298460	11388180	11300107	11374076	11330917
**NA50**	138423	125944	131985	138791	136025
**NGA50**	125189	123091	123976	126009	124495
**NA75**	86227	78078	78825	86815	81286
**NGA75**	69522	67666	67712	69022	67798
**LA50**	26	28	27	26	27

**LGA50**	29	31	30	29	29
**LA75**	52	57	55	52	54
**LGA75**	58	63	62	59	60

Staphylococcus aureus

Supplementary Table 1-30 Assembly statistics of gap filler on the Staphylococcus aureus dataset. All statistics are based on contigs of size≥500 bp, unless otherwise noted (e.g., "# contigs(≥0 bp)" and "Total length (≥0 bp)" include all contigs).

**Assembly**	**Scaffold**	**GapCloser**	**RFfiller**	**Sealer**	**TGS-GapCloser**
**# contigs (>= 0 bp)**	180	180	180	180	180
**# contigs (>= 1000 bp)**	46	46	46	46	46
**# contigs (>= 5000 bp)**	37	37	37	37	37
**# contigs (>= 10000 bp)**	34	34	34	34	34
**# contigs (>= 25000 bp)**	28	28	28	28	28
**# contigs (>= 50000 bp)**	18	18	18	18	18
**Total length (>= 0 bp)**	2779346	2787096	2779346	2784436	2790751
**Total length (>= 1000 bp)**	2756125	2763874	2756125	2761044	2767530
**Total length (>= 5000 bp)**	2732555	2739812	2732555	2737000	2743960
**Total length (>= 10000 bp)**	2707432	2714583	2707432	2711843	2718837
**Total length (>= 25000 bp)**	2608355	2613388	2608355	2611520	2619714
**Total length (>= 50000 bp)**	2232455	2236731	2232455	2235089	2235710
**# contigs**	55	55	55	55	55
**Largest contig**	353033	353524	353033	353773	355036
**Total length**	2762358	2770107	2762358	2767277	2773763
**Reference length**	2821361	2821361	2821361	2821361	2821361
**GC (%)**	32.64	32.66	32.64	32.66	32.64
**Reference GC (%)**	32.87	32.87	32.87	32.87	32.87
**N50**	127233	127201	127233	127201	127338
**NG50**	127233	127201	127233	127201	127338
**N75**	59777	59982	59777	59899	59826

**NG75**	59777	59982	59777	59899	59826
**L50**	7	7	7	7	7
**LG50**	7	7	7	7	7
**L75**	16	16	16	16	16
**LG75**	16	16	16	16	16
**# misassemblies**	35	45	34	45	35
**# misassembled contigs**	21	22	21	22	21
**Misassembled contigs length**	2171117	2193833	2171117	2191828	2182378
**# local misassemblies**	67	85	80	82	71
**# scaffold gap ext. mis.**	3	0	3	0	2
**# scaffold gap loc. mis.**	29	0	20	8	21
**# unaligned mis. contigs**	3	3	2	2	3
**# unaligned contigs**	6 + 30 part	6 + 29 part	6 + 29 part	6 + 29 part	6 + 30 part
**Unaligned length**	266649	266213	265533	265724	270587
**Genome fraction (%)**	88.254	88.507	88.285	88.454	88.514
**Duplication ratio**	1.002	1.003	1.002	1.002	1.002
**# N's per 100 kbp**	128.19	0.54	69.58	25.69	106.1
**# mismatches per 100 kbp**	1385.49	1390.66	1393.1	1389.47	1398.31
**# indels per 100 kbp**	49.52	48.66	51.59	48.28	62.79
**Largest alignment**	264638	171163	264638	171163	171108
**Total aligned length**	2490259	2500781	2491153	2497918	2497805
**NA50**	72552	72374	72552	72374	72623
**NGA50**	72552	72374	72552	72374	72623
**NA75**	30613	22215	30613	22215	32746
**NGA75**	22340	21757	22340	21757	30613
**LA50**	12	14	12	14	13
**LGA50**	12	14	12	14	13
**LA75**	28	32	28	32	29
**LGA75**	30	33	30	33	30

SPAdes

Arabidopsis thaliana

Supplementary Table 1-31 Assembly statistics of gap filler on the Arabidopsis thaliana dataset. All statistics are based on contigs of size≥500 bp, unless otherwise noted (e.g., "# contigs(≥0 bp)" and "Total length (≥0 bp)" include all contigs).

Assembly	Scaffold	GapCloser	RFfiller	Sealer	TGS-GapCloser
**# contigs (>= 0 bp)**	117150	117150	117150	117150	117150
**# contigs (>= 1000 bp)**	3123	3123	3123	3123	3123
**# contigs (>= 5000 bp)**	46	46	46	46	46
**# contigs (>= 10000 bp)**	18	18	18	18	18
**# contigs (>= 25000 bp)**	2	2	2	2	2
**# contigs (>= 50000 bp)**	0	0	0	0	0
**Total length (>= 0 bp)**	49357917	49357918	49357917	49357917	49357917
**Total length (>= 1000 bp)**	4699610	4699610	4699610	4699610	4699610
**Total length (>= 5000 bp)**	519750	519750	519750	519750	519750
**Total length (>= 10000 bp)**	324109	324109	324109	324109	324109
**Total length (>= 25000 bp)**	83768	83768	83768	83768	83768
**Total length (>= 50000 bp)**	0	0	0	0	0
**# contigs**	26750	26750	26750	26750	26750
**Largest contig**	43006	43006	43006	43006	43006
**Total length**	20218565	20218566	20218565	20218565	20218565
**Reference length**	119668634	119668634	119668634	119668634	119668634
**GC (%)**	36.48	36.48	36.48	36.48	36.48
**Reference GC (%)**	36.06	36.06	36.06	36.06	36.06
**N50**	723	723	723	723	723
**N75**	590	590	590	590	590
**L50**	9628	9628	9628	9628	9628
**L75**	17412	17412	17412	17412	17412

**# misassemblies**	7944	7944	7944	7944	7944
**# misassembled contigs**	6761	6761	6761	6761	6761
**Misassembled contigs length**	6029739	6029740	6029739	6029739	6029739
**# local misassemblies**	553	553	555	553	553
**# scaffold gap ext. mis.**	0	0	0	0	0
**# scaffold gap loc. mis.**	3	4	3	3	3
**# unaligned mis. contigs**	95	95	95	95	95
**# unaligned contigs**	232 + 178 part	232 + 178 part	232 + 178 part	232 + 178 part	232 + 178 part
**Unaligned length**	311223	311223	311223	311223	311223
**Genome fraction (%)**	15.986	15.987	15.986	15.986	15.986
**Duplication ratio**	1.042	1.042	1.042	1.042	1.042
**# N's per 100 kbp**	1.58	1.19	0.59	1.58	1.58
**# mismatches per 100 kbp**	824.1	824.29	824.11	824.1	824.1
**# indels per 100 kbp**	67.08	67.07	67.07	67.08	67.08
**Largest alignment**	42137	42137	42137	42137	42137
**Total aligned length**	19102067	19102325	19101872	19102067	19102067
**NA50**	606	606	606	606	606
**NGA50**	-	-	-	-	-
**NA75**	501	501	501	501	501
**LA50**	11850	11850	11850	11850	11850
**LA75**	21073	21073	21074	21073	21073

Bacillus cereus

Supplementary Table 1-32 Assembly statistics of gap filler on the Bacillus cereus dataset. All statistics are based on contigs of size≥500 bp, unless otherwise noted (e.g., "# contigs(≥0 bp)" and "Total length (≥0 bp)" include all contigs).

**Assembly**	**Scaffold**	**GapCloser**	**RFfiller**	**Sealer**	**TGS-GapCloser**
**# contigs (>= 0 bp)**	220	220	220	220	220
**# contigs (>= 1000 bp)**	119	119	119	119	119

**# contigs (>= 5000 bp)**	99	99	99	99	99
**# contigs (>= 10000 bp)**	81	81	81	81	81
**# contigs (>= 25000 bp)**	51	51	51	51	51
**# contigs (>= 50000 bp)**	34	34	34	34	34
**Total length (>= 0 bp)**	5421489	5421416	5421489	5420812	5421489
**Total length (>= 1000 bp)**	5384454	5384381	5384454	5383777	5384454
**Total length (>= 5000 bp)**	5335066	5334992	5335066	5334389	5335066
**Total length (>= 10000 bp)**	5214318	5214244	5214318	5213641	5214318
**Total length (>= 25000 bp)**	4747699	4747625	4747699	4747022	4747699
**Total length (>= 50000 bp)**	4132854	4132780	4132854	4132177	4132854
**# contigs**	138	138	138	138	138
**Largest contig**	421518	421519	421518	421418	421518
**Total length**	5396320	5396247	5396320	5395643	5396320
**Reference length**	5427083	5427083	5427083	5427083	5427083
**GC (%)**	35.34	35.34	35.34	35.34	35.34
**Reference GC (%)**	35.29	35.29	35.29	35.29	35.29
**N50**	94674	94597	94674	94474	94674
**NG50**	94674	94597	94674	94474	94674
**N75**	53460	53460	53460	53460	53460
**NG75**	53460	53460	53460	53460	53460
**L50**	14	14	14	14	14
**LG50**	14	14	14	14	14
**L75**	33	33	33	33	33
**LG75**	33	33	33	33	33
**# misassemblies**	0	0	0	0	0
**# misassembled contigs**	0	0	0	0	0
**Misassembled contigs length**	0	0	0	0	0
**# local misassemblies**	4	4	4	4	4
**# scaffold gap ext. mis.**	0	0	0	0	0
**# scaffold gap loc. mis.**	0	0	0	0	0
**# unaligned mis. contigs**	42	42	42	42	42

# unaligned contigs	66 + 67 part	66 + 67 part	66 + 67 part	66 + 67 part	66 + 67 part
Unaligned length	5088204	5089024	5088204	5088280	5088204
Genome fraction (%)	5.629	5.625	5.629	5.628	5.629
Duplication ratio	1.009	1.006	1.009	1.006	1.009
# N's per 100 kbp	14.82	1.95	12.97	3.71	14.82
# mismatches per 100 kbp	3763.1	3765.25	3763.43	3762.48	3763.1
# indels per 100 kbp	32.08	32.43	34.04	32.09	32.08
Largest alignment	31143	31143	31143	31143	31143
Total aligned length	307416	307119	307416	307263	307416
NGA50	-	-	-	-	-

Escherichia coli

Supplementary Table 1-33 Assembly statistics of gap filler on the Escherichia coli dataset. All statistics are based on contigs of size≥500 bp, unless otherwise noted (e.g., "# contigs(≥0 bp)" and "Total length (≥0 bp)" include all contigs).

Assembly	Scaffold	GapCloser	RFfiller	Sealer	TGS-GapCloser
# contigs (>= 0 bp)	591	591	591	591	591
# contigs (>= 1000 bp)	166	166	166	166	166
# contigs (>= 5000 bp)	85	85	85	85	85
# contigs (>= 10000 bp)	64	64	64	64	64
# contigs (>= 25000 bp)	51	51	51	51	51
# contigs (>= 50000 bp)	27	27	27	27	27
Total length (>= 0 bp)	5598648	5598652	5598648	5598348	5598137
Total length (>= 1000 bp)	5423881	5423885	5423881	5423581	5423370
Total length (>= 5000 bp)	5228281	5228284	5228281	5227981	5227770
Total length (>= 10000 bp)	5076285	5076287	5076285	5075985	5075774
Total length (>= 25000 bp)	4836815	4836817	4836815	4836515	4836304
Total length (>= 50000 bp)	3968105	3968106	3968105	3967905	3967594
# contigs	274	274	274	274	274

**Largest contig**	351147	351147	351147	351147	351147
**Total length**	5497157	5497161	5497157	5496857	5496646
**Reference length**	4641652	4641652	4641652	4641652	4641652
**GC (%)**	50.2	50.2	50.2	50.2	50.2
**Reference GC (%)**	50.79	50.79	50.79	50.79	50.79
**N50**	128635	128635	128635	128635	128635
**NG50**	142593	142593	142593	142593	142593
**N75**	43645	43645	43645	43645	43645
**NG75**	86355	86355	86355	86355	86355
**L50**	13	13	13	13	13
**LG50**	10	10	10	10	10
**L75**	31	31	31	31	31
**LG75**	21	21	21	21	21
**# misassemblies**	99	99	99	99	99
**# misassembled contigs**	39	39	39	39	39
**Misassembled contigs length**	4148838	4148839	4148838	4148638	4148327
**# local misassemblies**	148	149	149	149	148
**# scaffold gap ext. mis.**	0	0	0	0	0
**# scaffold gap loc. mis.**	2	1	1	1	1
**# unaligned mis. contigs**	11	11	11	11	11
**# unaligned contigs**	170 + 84 part	170 + 84 part	170 + 84 part	170 + 84 part	170 + 84 part
**Unaligned length**	1405380	1405598	1405480	1405286	1405192
**Genome fraction (%)**	87.713	87.716	87.713	87.713	87.713
**Duplication ratio**	1.005	1.005	1.005	1.005	1.005
**# N's per 100 kbp**	9.1	0.96	5.46	3.64	5.46
**# mismatches per 100 kbp**	1653.93	1654.04	1653.93	1654.08	1654.15
**# indels per 100 kbp**	23.26	23.28	23.26	23.24	23.31
**Largest alignment**	335305	335305	335305	335305	335305
**Total aligned length**	4083962	4084096	4083962	4083956	4083750
**NA50**	31972	31972	31972	31972	31972

**NGA50**	36694	36694	36694	36694	36694
**NGA75**	15999	15999	15999	15999	15999
**LA50**	44	44	44	44	44
**LGA50**	32	32	32	32	32
**LGA75**	75	75	75	75	75

Homo sapien

Supplementary Table 1-34 Assembly statistics of gap filler on the Homo sapien dataset. All statistics are based on contigs of size≥500 bp, unless otherwise noted (e.g., "# contigs(≥0 bp)" and "Total length (≥0 bp)" include all contigs).

**Assembly**	**Scaffold**	**GapCloser**	**RFfiller**	**Sealer**	**TGS-GapCloser**
**# contigs (>= 0 bp)**	1592	1592	1592	1592	1592
**# contigs (>= 1000 bp)**	72	72	72	72	72
**# contigs (>= 5000 bp)**	2	2	2	2	10
**# contigs (>= 10000 bp)**	1	1	1	1	3
**# contigs (>= 25000 bp)**	0	0	0	0	0
**# contigs (>= 50000 bp)**	0	0	0	0	0
**Total length (>= 0 bp)**	346218	346390	346218	346218	399913
**Total length (>= 1000 bp)**	158300	158449	158300	158300	212041
**Total length (>= 5000 bp)**	22564	22564	22564	22564	90190
**Total length (>= 10000 bp)**	16600	16600	16600	16600	37472
**Total length (>= 25000 bp)**	0	0	0	0	0
**Total length (>= 50000 bp)**	0	0	0	0	0
**# contigs**	134	134	134	134	134
**Largest contig**	16600	16600	16600	16600	16600
**Total length**	200396	200564	200396	200396	254091
**Reference length**	3272089205	3272089205	3272089205	3272089205	3272089205
**GC (%)**	50.91	50.89	50.91	50.91	50.71
**Reference GC (%)**	41.04	41.04	41.04	41.04	41.04
**N50**	1882	1882	1882	1882	3224

**N75**	1101	1102	1101	1101	1275
**L50**	30	30	30	30	19
**L75**	65	65	65	65	53
**# misassemblies**	106	118	119	106	100
**# misassembled contigs**	66	72	74	66	63
**Misassembled contigs length**	88339	99616	101566	88339	102443
**# local misassemblies**	12	16	17	12	17
**# scaffold gap ext. mis.**	17	3	1	17	10
**# scaffold gap loc. mis.**	2	1	0	2	1
**# unaligned mis. contigs**	3	3	3	3	2
**# unaligned contigs**	11 + 6 part	11 + 6 part	11 + 6 part	11 + 6 part	11 + 6 part
**Unaligned length**	18523	18559	18611	18523	22538
**Genome fraction (%)**	0.006	0.006	0.006	0.006	0.007
**Duplication ratio**	1.058	1.051	1.057	1.058	1.07
**# N's per 100 kbp**	931.66	356	166.17	931.66	660
**# mismatches per 100 kbp**	1392.7	1382.99	1371.07	1392.7	1454.35
**# indels per 100 kbp**	65.18	62.94	62.77	65.18	113.26
**Largest alignment**	16600	16600	16600	16600	16600
**Total aligned length**	171986	173379	172195	171986	221963
**NA50**	880	840	827	880	1546
**NGA50**	-	-	-	-	-
**NA75**	324	324	306	324	448
**LA50**	47	51	52	47	28
**LA75**	137	145	148	137	105

Saccharomyces cerevisiae

Supplementary Table 1-35 Assembly statistics of gap filler on the Saccharomyces cerevisiae dataset. All statistics are based on contigs of size≥500 bp, unless otherwise noted (e.g., ”# contigs(≥0 bp)” and ”Total length (≥0 bp)” include all contigs).

Assembly	Scaffold	GapCloser	RFfiller	Sealer	TGS-GapCloser
**# contigs (>= 0 bp)**	2295	2295	2295	2295	2295
**# contigs (>= 1000 bp)**	216	217	216	216	216
**# contigs (>= 5000 bp)**	158	158	158	158	158
**# contigs (>= 10000 bp)**	141	141	141	141	141
**# contigs (>= 25000 bp)**	113	113	113	113	113
**# contigs (>= 50000 bp)**	82	82	82	82	82
**Total length (>= 0 bp)**	11871908	11871331	11871908	11865215	11865867
**Total length (>= 1000 bp)**	11473203	11473577	11473203	11466810	11467162
**Total length (>= 5000 bp)**	11357435	11356641	11357435	11351283	11351507
**Total length (>= 10000 bp)**	11239996	11239201	11239996	11233937	11234068
**Total length (>= 25000 bp)**	10780243	10779446	10780243	10774474	10774619
**Total length (>= 50000 bp)**	9602639	9601817	9602639	9597822	9597926
**# contigs**	313	313	313	313	313
**Largest contig**	381132	381132	381132	381132	381132
**Total length**	11537298	11536720	11537298	11530705	11531257
**Reference length**	12157105	12157105	12157105	12157105	12157105
**GC (%)**	38.14	38.14	38.14	38.14	38.14
**Reference GC (%)**	38.15	38.15	38.15	38.15	38.15
**N50**	114579	114580	114579	114424	114424
**NG50**	109730	109730	109730	109730	109730
**N75**	71275	71275	71275	71275	71275
**NG75**	60166	60166	60166	60166	60166
**L50**	35	35	35	35	35
**LG50**	38	38	38	38	38
**L75**	66	66	66	66	66
**LG75**	74	74	74	74	74
**# misassemblies**	59	59	59	57	57
**# misassembled contigs**	44	44	44	42	42

**Misassembled contigs length**	3606168	3606090	3606168	3508292	3508291
**# local misassemblies**	101	132	114	102	100
**# scaffold gap ext. mis.**	0	0	0	0	0
**# scaffold gap loc. mis.**	55	6	40	1	3
**# unaligned mis. contigs**	2	2	2	2	2
**# unaligned contigs**	58 + 23 part	58 + 23 part	58 + 23 part	58 + 23 part	58 + 23 part
**Unaligned length**	134977	135032	135174	134359	134613
**Genome fraction (%)**	93.535	93.544	93.541	93.539	93.537
**Duplication ratio**	1.003	1.003	1.003	1.002	1.002
**# N's per 100 kbp**	67.08	12.64	47.14	7.38	10.74
**# mismatches per 100 kbp**	579.78	580.74	581	580.34	581.25
**# indels per 100 kbp**	49.2	49.45	49.89	49.41	50.93
**Largest alignment**	328316	328427	328316	328426	328924
**Total aligned length**	11379138	11382745	11379596	11377399	11377044
**NA50**	102454	102454	102454	102454	102129
**NGA50**	93828	93828	93828	93828	93828
**NA75**	54784	54784	54784	54760	54744
**NGA75**	45331	45331	45331	45331	45331
**LA50**	41	41	41	41	41
**LGA50**	44	44	44	44	44
**LA75**	79	79	79	79	79
**LGA75**	89	89	89	89	89

Staphylococcus aureus

Supplementary Table 1-36 Assembly statistics of gap filler on the Staphylococcus aureus dataset. All statistics are based on contigs of size≥500 bp, unless otherwise noted (e.g., "# contigs(≥0 bp)" and "Total length (≥0 bp)" include all contigs).

**Assembly**	**Scaffold**	**GapCloser**	**RFfiller**	**Sealer**	**TGS-GapCloser**
**# contigs (>= 0 bp)**	79	79	79	79	79

**# contigs (>= 1000 bp)**	33	33	33	33	33
**# contigs (>= 5000 bp)**	25	25	25	25	25
**# contigs (>= 10000 bp)**	24	24	24	24	24
**# contigs (>= 25000 bp)**	21	21	21	21	21
**# contigs (>= 50000 bp)**	16	16	16	16	16
**Total length (>= 0 bp)**	2797545	2797411	2797545	2796945	2798248
**Total length (>= 1000 bp)**	2780900	2780766	2780900	2780300	2781603
**Total length (>= 5000 bp)**	2762168	2762033	2762168	2761568	2762871
**Total length (>= 10000 bp)**	2754268	2754133	2754268	2753668	2754971
**Total length (>= 25000 bp)**	2697403	2697267	2697403	2696903	2698106
**Total length (>= 50000 bp)**	2496997	2496860	2496997	2496597	2497700
**# contigs**	40	40	40	40	40
**Largest contig**	406941	406942	406941	406841	406565
**Total length**	2784943	2784809	2784943	2784343	2785646
**Reference length**	2821361	2821361	2821361	2821361	2821361
**GC (%)**	32.66	32.66	32.66	32.66	32.67
**Reference GC (%)**	32.87	32.87	32.87	32.87	32.87
**N50**	174162	174162	174162	174162	174162
**NG50**	174162	174162	174162	174162	174162
**N75**	108187	108187	108187	108187	108187
**NG75**	108187	108187	108187	108187	108187
**L50**	6	6	6	6	6
**LG50**	6	6	6	6	6
**L75**	11	11	11	11	11
**LG75**	11	11	11	11	11
**# misassemblies**	54	54	54	54	54
**# misassembled contigs**	16	16	16	16	16
**Misassembled contigs length**	2442882	2442745	2442882	2442482	2443585
**# local misassemblies**	90	94	93	92	93
**# scaffold gap ext. mis.**	0	0	0	0	0
**# scaffold gap loc. mis.**	4	0	1	1	2

**# unaligned mis. contigs**	4	4	4	4	4
**# unaligned contigs**	7 + 22 part	7 + 22 part	7 + 22 part	7 + 22 part	7 + 22 part
**Unaligned length**	277231	276651	277362	276066	277755
**Genome fraction (%)**	88.605	88.624	88.605	88.627	88.614
**Duplication ratio**	1.003	1.003	1.003	1.003	1.003
**# N's per 100 kbp**	28.73	0.22	10.77	7.22	14.36
**# mismatches per 100 kbp**	1390.88	1392.42	1391.72	1392.32	1391.42
**# indels per 100 kbp**	48.08	48.39	48.44	48.47	49.24
**Largest alignment**	171163	171163	171163	171163	171163
**Total aligned length**	2505064	2505702	2505133	2506029	2505443
**NA50**	72014	72014	72014	72014	72014
**NGA50**	72014	72014	72014	72014	72014
**NA75**	23621	23621	23621	23621	23621
**NGA75**	22340	22340	22340	22340	22340
**LA50**	13	13	13	13	13
**LGA50**	13	13	13	13	13
**LA75**	30	30	30	30	30
**LGA75**	31	31	31	31	31

Chapter 2 Scripts

GapCloser

Configuration file

#maximal read length
max_rd_len=<maximum_read_length_of_read>
[LIB]
#average insert size of the library
avg_ins=<average_inser_size_of_library>
#if sequences are forward-reverse of reverse-forward
reverse_seq=0
#in which part(s) the reads are used (only contigs, only scaffolds, both contigs and scaffolds, only gap closure)
asm_flags=3

#cut the reads to the given length
rd_len_cutoff=100
#in which order the reads are used while scaffolding
rank=1
cutoff of pair number for a reliable connection (at least 3 for short insert size)
pair_num_cutoff=3
#minimum aligned length to contigs for a reliable read location (at least 32 for short insert size)
map_len=32
#fastq file for single reads
q1=<path_of_read_1>
q2=<path_of_read_2>

Script

GapCloser -a <path_of_scaffold> -b <path_of_config> -o <output> -t 40

Sealer

abyss-sealer -b500M -k90 -k80 -k70 -k60 -k50 -k40 -k30 -j 40 -o bc -S <path_of_scaffold> <path_of_read_1> <path_of_read_2>

TGS-GapCloser

tgsgapcloser --scaff <path_of_scaffold> --reads <path_of_read> --output <output> --thread 40 --ne

Rffiller

rffiller.py --scaf <path_of_scaffold> --long_read <path_of_read> --output <output>

Chapter 3 Summary

De novo assembly

Table 3-1 Summarized de novo assembly statistics across all datasets. TAL refers to Total Aligned Length

Assembler	Dataset	gaps	misassembly	mismatch	TAL	NGA50
**A5-MiSeq**	Arabidopsis thaliana	63610	352	33310	7815659	-
	Bacillus cereus	799	1	11939	321616	-
	Escherichia coli	558	99	67504	4096071	36307
	Homo sapiens	697917	5393	177164	75974294	-
	Saccharomyces cerevisiae	40	71	63788	11423212	72006

	Staphylococcus aureus	6137	67	24937	2518210	72014
**ABySS**	Arabidopsis thaliana	0	12	1274	648770	-
	Bacillus cereus	1467	1	11700	306444	-
	Escherichia coli	1117	82	66967	4098089	32234
	Homo sapiens	0	0	97	42834	-
	Saccharomyces cerevisiae	215	64	65892	11408087	92224
	Staphylococcus aureus	5077	42	34627	2495696	55411
**FLYE**	Arabidopsis thaliana	200	2562	1351786	87766020	31486
	Bacillus cereus	0	5	34873	988081	-
	Escherichia coli	0	182	92861	4204188	51984
	Homo sapiens	0	749	690191	53251209	-
	Saccharomyces cerevisiae	0	0	14	531	-
	Staphylococcus aureus	300	54	5019	2617887	92541
**SGA**	Arabidopsis thaliana	0	0	278	359572	-
	Bacillus cereus	900	1	12486	342625	-
	Escherichia coli	0	52	68003	4193611	6669
	Homo sapiens	0	0	703	154701	-
	Saccharomyces cerevisiae	5200	70	66403	11428586	121483
	Staphylococcus aureus	14376	51	33724	2521599	35516
**SOAPdenovo2**	Arabidopsis thaliana	3137	8	304	299308	-
	Bacillus cereus	5221	1	12468	321970	-
	Escherichia coli	4238	41	64503	4025781	8412
	Homo sapiens	173917	40	3187	566688	-
	Saccharomyces cerevisiae	3541	48	63719	11298460	125189
	Staphylococcus aureus	41059	35	34498	2490259	72552
**SPAdes**	Arabidopsis thaliana	320	7944	157411	19102067	-
	Bacillus cereus	800	0	11496	307416	-
	Escherichia coli	500	99	67337	4083962	36694
	Homo sapiens	1867	106	2393	171986	-
	Saccharomyces cerevisiae	800	59	65928	11379138	93828
	Staphylococcus aureus	7739	54	34770	2505064	72014

Gap fillers

Table 3-2 Summary of all gap fillers statistics across all datasets. TAL refers to Total Aligned Length.

Assemblers	Gap filling algorithms	Arabidopsis thaliana					Bacillus cereus					Escherichia coli				
		# gaps	Misassembly	Mismatch	TAL	NGA50	# gaps	Misassembly	Mismatch	TAL	NGA50	# gaps	Misassembly	Mismatch	TAL	NGA50
A5-MiSeq	Proposed algorithm	63610	342	32301	7815676	-	799	1	11919	321776	-	558	99	67500	4096078	36307
	GapCloser	0	376	57482	7586395	-	0	1	11942	321724	-	0	99	67631	4096071	36307
	Sealer	63390	350	32978	7802685	-	791	1	11945	321757	-	557	99	67503	4096049	36307
	TGS-GapCloser	63610	352	33310	7815659	-	799	1	11939	321616	-	558	99	67504	4096071	36307
ABySS	Proposed algorithm	0	12	1274	648770	-	1164	1	11681	306960	-	829	80	66941	4098667	31906
	GapCloser	0	12	1274	648770	-	612	1	11716	306853	-	165	84	66977	4098461	31906
	Sealer	0	12	1274	648770	-	678	1	11706	306638	-	399	83	66986	4098645	31906
	TGS-GapCloser	0	12	1274	648770	-	1367	1	11700	306444	-	915	82	67015	4100013	32234
FLYE	Proposed algorithm	200	2562	1351786	87766020	31486	0	5	34873	988081	-	0	182	92861	4204188	51984
	GapCloser	200	2562	1351786	87766020	31486	0	5	34873	988081	-	0	182	92861	4204188	51984
	Sealer	200	2562	1351786	87766020	31486	0	5	34873	988081	-	0	182	92861	4204188	51984

	**TGS-GapCloser**	200	2562	1351786	87766020	31486	0	5	34873	988081	-	0	182	92861	4204188	51984
**SGA**	**Proposed algorithm**	0	0	278	359572	-	850	1	12478	343685	-	0	52	68003	4193611	5205
	**GapCloser**	0	0	278	359572	-	36	1	12488	342564	-	0	52	68003	4193611	5205
	**Sealer**	0	0	278	359572	-	900	1	12486	342625	-	0	52	68003	4193611	5205
	**TGS-GapCloser**	0	0	278	359572	-	725	1	12541	343657	-	0	52	68003	4193611	5205
**SOAPdenovo2**	**Proposed algorithm**	2374	6	368	299496	-	4435	1	12468	322133	-	2895	40	64489	4037326	8463
	**GapCloser**	199	5	320	300945	-	191	1	12484	322683	-	135	43	64737	4032246	8372
	**Sealer**	257	4	328	303865	-	364	1	12436	322378	-	431	42	64918	4036849	8441
	**TGS-GapCloser**	2240	8	371	299936	-	5134	1	12468	321963	-	3845	41	64561	4027136	8441
**SPAdes**	**Proposed algorithm**	120	7944	157212	19102872	-	700	0	11490	307461	-	300	99	67337	4084262	31972
	**GapCloser**	240	7944	157450	19102325	-	105	0	11494	307119	-	53	99	67344	4084096	31972
	**Sealer**	320	7944	157411	19102067	-	200	0	11491	307263	-	200	99	67343	4083956	31972
	**TGS-GapCloser**	320	7944	157411	19102067	-	800	0	11496	307416	-	300	99	67346	4083750	31972
		**Homo sapiens**					**Saccharomyces cerevisiae**					**Staphylococcus aureus**				
**A5-MiSeq**	**Proposed algorithm**	697799	5372	175157	75974256	-	5687	69	63796	11424212	72006	40	67	34927	2518210	72014

	**Gap Closer**	90	5955	525161	74787610	-	16	68	67673	11423302	72006	0	67	34949	2518210	72014
	**Sealer**	697213	5381	176575	75904734	-	6005	70	63868	11423779	72006	40	67	34935	2517945	72014
	**TGS-Gap Closer**	697730	5393	177170	75974192	-	5769	70	63830	11424071	72006	40	67	34937	2518210	72014
**ABySS**	**Proposed algorithm**	0	0	97	42834	-	3483	60	65965	11418091	92324	160	42	34627	2496896	55411
	**Gap Closer**	0	0	97	42834	-	324	67	65980	11410118	92225	9	42	34630	2495826	55411
	**Sealer**	0	0	97	42834	-	1708	64	65970	11411158	92305	2	42	34631	2495696	55411
	**TGS-Gap Closer**	0	0	97	42834	-	940	67	66225	11412329	89077	55	44	34662	2496097	55411
**FLYE**	**Proposed algorithm**	0	749	690191	53251209	-	300	0	14	531	-	0	54	5019	2617887	92541
	**Gap Closer**	0	749	690191	53251209	-	200	0	1	269	-	0	54	5019	2617887	92541
	**Sealer**	0	749	690191	53251209	-	300	0	14	531	-	0	54	5019	2617887	92541
	**TGS-Gap Closer**	0	749	690191	53251209	-	300	0	14	531	-	0	54	5019	2617887	92541
**SGA**	**Proposed algorithm**	0	0	703	154701	-	7300	62	66486	11448604	121483	3450	47	33620	2525510	36119
	**Gap Closer**	0	0	703	154701	-	463	83	66914	11440151	121483	203	54	33787	2525370	35541
	**Sealer**	0	0	703	154701	-	9003	68	66632	11432292	118186	5108	51	33724	2521599	35516
	**TGS-Gap**	0	0	703	154701	-	5266	79	66814	11431473	114505	1975	50	34641	2515950	36053

	**Closer**															
**SOAPdenovo2**	**Proposed algorithm**	2521	42	21040	442912	-	25851	55	64196	11340071	123976	1922	37	34700	2491153	72552
	**Gap Closer**	9211	26	4396	522892	-	549	71	65214	11388180	123091	15	45	34726	2500781	72374
	**Sealer**	173917	40	3187	566688	-	11358	54	65039	11374076	126009	711	45	34676	2497918	72374
	**TGS-Gap Closer**	171396	39	3249	570729	-	36311	58	65174	11330917	124495	2943	35	34920	2497918	72623
**SPAdes**	**Proposed algorithm**	333	100	2359	172195	-	5439	56	66064	11389730	93828	300	54	34761	2506133	72014
	**Gap Closer**	714	118	2395	173379	-	1458	59	66043	11382745	93828	6	54	34816	2505702	72014
	**Sealer**	1867	106	2393	171986	-	851	57	65994	11377399	93828	201	54	34815	2506029	72014
	**TGS-Gap Closer**	1677	100	3146	221963	-	1239	57	66096	11377044	93828	400	54	34787	2505443	72014
